# Dynamic cancer drivers: a causal approach for cancer driver discovery based on bio-pathological trajectories

**DOI:** 10.1093/bfgp/elac030

**Published:** 2022-09-19

**Authors:** Andres M Cifuentes-Bernal, Vu V H Pham, Xiaomei Li, Lin Liu, Jiuyong Li, Thuc Duy Le

**Affiliations:** UniSA STEM Unit, University of South Australia, Mawson Lakes Blvd, 5095, South Australia , Australia; UniSA STEM Unit, University of South Australia, Mawson Lakes Blvd, 5095, South Australia , Australia; UniSA STEM Unit, University of South Australia, Mawson Lakes Blvd, 5095, South Australia , Australia; UniSA STEM Unit, University of South Australia, Mawson Lakes Blvd, 5095, South Australia , Australia; UniSA STEM Unit, University of South Australia, Mawson Lakes Blvd, 5095, South Australia , Australia; UniSA STEM Unit, University of South Australia, Mawson Lakes Blvd, 5095, South Australia , Australia

**Keywords:** Driver genes, dynamics, causality, pseudotime, causal impact, cancer

## Abstract

The traditional way for discovering genes which drive cancer (namely cancer drivers) neglects the dynamic information of cancer development, even though it is well known that cancer progresses dynamically. To enhance cancer driver discovery, we expand cancer driver concept to dynamic cancer driver as a gene driving one or more bio-pathological transitions during cancer progression. Our method refers to the fact that cancer should not be considered as a single process but a compendium of altered biological processes causing the disease to develop over time. Reciprocally, different drivers of cancer can potentially be discovered by analysing different bio-pathological pathways. We propose a novel approach for causal inference of genes driving one or more core processes during cancer development (i.e. dynamic cancer driver). We use the concept of pseudotime for inferring the latent progression of samples along a biological transition during cancer and identifying a critical event when such a process is significantly deviated from normal to carcinogenic. We infer driver genes by assessing the causal effect they have on the process after such a critical event. We have applied our method to single-cell and bulk sequencing datasets of breast cancer. The evaluation results show that our method outperforms well-recognized cancer driver inference methods. These results suggest that including information of the underlying dynamics of cancer improves the inference process (in comparison with using static data), and allows us to discover different sets of driver genes from different processes in cancer. R scripts and datasets can be found at https://github.com/AndresMCB/DynamicCancerDriver

## 1 Introduction

Cancer development is traditionally linked to changes at genome level, commonly known as ‘driver’ mutations, that confer proliferative advantage to some cells over others, usually leading to a neoplasm [1]. Among the mutations that can be found in cancer tumours, a minimum number promotes tumourigenesis, especially in comparison with the vast number of passenger mutations that do not confer superiority to cancer cells [2]. For this reason, most efforts in recent decades have been focused on distinguishing between genes harbouring driver mutations and genes with mutations that do not provide selective advantages to the cell [3, 4].

The impact of a genetic aberration can be reflected on other genes besides the mutated gene as genes interact in complex biological networks [5, 6]. As a result, a mutated gene can affect gene products further than its own, even in genes carrying no defects [7]. Taking this into consideration, some methods of cancer drivers discovery have integrated biological network information [8, 9]. These approaches have become popular in recent years due to the fact they can integrate and summarize different layers of data in an easy and comprehensible way. A detailed review and comparison of cancer drivers discovery methods can be found in [10].

**Figure 1:**
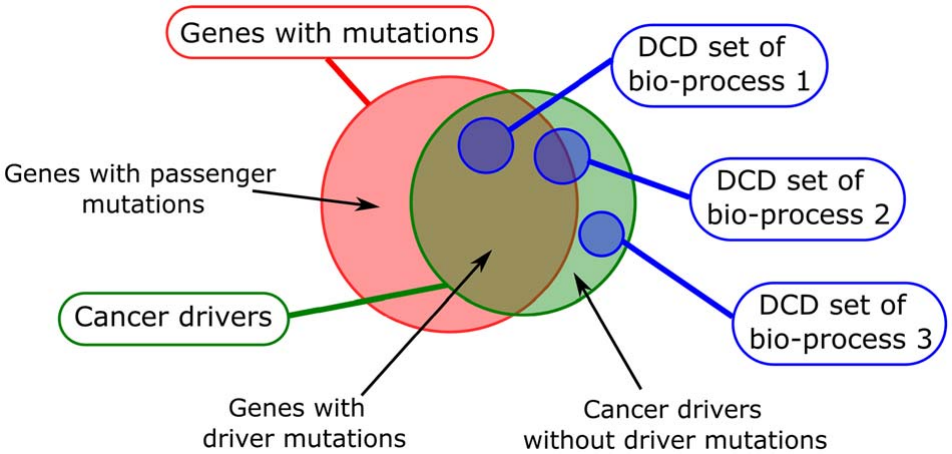
Graphical representation of the overlap of different categories of cancer drivers (adapted from [10]). Genes with driver mutations are cancer drivers. Recent findings have shown some genes without driver mutations can also drive cancer progression. Genes driving biological processes leading to cancer (i.e ***dynamic cancer drivers***, DCD for short) are not necessary mutated. Additionally, different biological core processes (shorten in the figure as bio-process) are driven by different set of genes.

The fundamental hypothesis in this paper is that ‘incorporating the dynamic aspect of biological processes driving cancer progression allows us to detect ***cancer drivers*** that are not identifiable by methods based on data that neglects the dynamic’, and neglecting dynamics during inference process reduces the spectrum of cancer drivers that can be detected from observational data. Formally, throughout this paper we name a gene driving one or more core processes over cancer progression as ***dynamic cancer driver*** (DCD for short). Genes driving such processes may not be mutated as not all cancer drivers harbour driver mutations (Figure 1, please see [10] for a review). Moreover, anomalies in biological processes leading to cancer development may occur at different stages of cancer progression, and thus the set of cancer drivers may not be a fixed set throughout the progression of the disease.

Our hypothesis is based on the fact that mutational and network-based approaches infer cancer drivers without using the dynamical aspect of the disease. Cancer, as well as a large number of other diseases, is a compendium of heterogeneous processes. Cancer is considered to be driven by irregularities (including mutations) at different molecular levels, such as genetic, epigenetic and transcriptomic levels [11]. Those irregularities alter normal biological processes. As a result, phenotypes linked to the disease are the consequence of various bio-pathological processes occurring within a complex network [7], evolving through time in a dynamical way.

Dynamical information (in the sense of information generated by a dynamic system) of some biological processes can be directly obtained from time series datasets (e.g. [12, 13]). But reliable disease-related real time series datasets are rare in practice as they are costly and technically difficult to get. Moreover, disease progression is influenced by a wide range of factors that cannot be completely replicated artificially. Additionally, most time series datasets for biological processes are obtained from controlled experiments in laboratories; they can usually represent accurately few processes of the disease progression at a time. For those reasons, the vast majority of cancer-related datasets are obtained from cross-sectional studies, where data (known as cross-sectional or static data) are collected at one single instant during the disease.

Capturing useful dynamical information from cross-sectional cancer data for drivers discovery becomes the key step for exploiting dynamic information in static data. The *pseudotime* concept, originally developed for single-cell data, is a good candidate for extracting dynamical information from biological processes. The basic idea of *pseudotime* is that given a collection of heterogeneous samples (usually single cells), disperse along a biological transition (a biological progression), each sample can be scored to reflect its stage in such a progression [14]. Recently, it has been found that, due to its intrinsically temporal nature, *pseudotime* can successfully extract aspects of the underlying dynamics of a biological process from both bulk and single-cell datasets [15–17].

To the best of our knowledge, three fundamental aspects should be considered during the inference of cancer drivers. Firstly, cancer evolves over time [18, 19], therefore it is a collection of dynamic processes and cancer drivers are actually driving one or more processes during cancer progression. Secondly, genomic aberrations that drive cancer progression are not limited to gene mutations [8, 20], so inference of cancer drivers based on gene mutational burden lacks information from other essential aspects of cancer evolution. Thirdly, under the definition of cancer driver as a gene that promotes the appearance and progression of cancer, a driver gene is part of the causes of cancer development. Consequently, the causal nature of the driver–cancer relationship should be considered during the inference process. To overcome the limitations of current mutational and network-based methods for cancer driver inference, while fulfilling the three fundamental aspects discussed above, we have developed a ***dynamic cancer drivers*** inference approach.

Our method takes gene expression data from cross-sectional studies, as well as a covariate that reasonably modulates (in the sense described by Campbell and Yau [15]) the *pseudotemporal* progression of one relevant process occurring during cancer development. If no *pseudotime* is provided, our method relies on *PhenoPath* [15, 21] to find a *pseudotime* score to order the samples following the trajectory encoded by the covariate. We use the *pseudotime* (either provided or inferred by using *PhenoPath*) and the covariate provided to find a critical turning point in the trajectory along the *pseudotime*. We name this critical point as the ‘event’ (further discussion in Section 2.3). Please note that in either case, our method requires a trajectory covariate.

We hypothesize that the causal relationship between a driver gene and cancer development induces a significant deviation (also referred as *Causal Impact*) of a core process from normal to carcinogenic after the ‘event’. This *Causal Impact* can be detected in the gene expression of such a driver gene. Thus, we assess the *Causal Impact* of each ‘relevant’ gene after the ‘event’ (details in Section 2). If a gene expression reflects a significant *Causal Impact* after the ‘event’, the corresponding gene is considered as cancer driver for our method.

We applied our ***dynamic cancer drivers*** approach to a single-cell RNA sequencing dataset (NCBI GEO database, accession GSE75688) [22], and *the cancer genome atlas* breast cancer dataset (TCGA BRCA) [23] (details in Sections 3.1 and 3.4, respectively). We assessed the ability of our method to expand current cancer drivers catalogues, as well as its inference power. We used The Cancer Gene Census (CGC) [24] as a conservative approximation of the ground truth since CGC catalogue is still in expansion, and thus non-CGC genes discovered by our method should not be ruled out as drivers. Additionally, we used the TCGA–BRCA data to compare our method with seven popular methods for cancer driver inference. The results show our method outperforms them as it can discover more CGC genes, with better precision than the other assessed methods.

Our experiments suggest our method can identify driver genes for different trajectories from the same dataset. Unlike other methods that can only identify a global (static) set of cancer drivers, our method discovers ***dynamic drivers***, i.e genes driving one or more core processes over cancer progression. Our results also suggest that the temporal information not only improves performance of cancer drivers inference but also allow us to identify genes driving specific processes along cancer development.

## 2 Methods

### 2.1 Problem definition and method overview

In our approach we name a ***dynamic cancer driver*** as a gene driving one (or more) significant biological processes along cancer progression. Under this definition, our approach aims to elucidate cancer drivers by incorporating to the inference process the dynamical information of the disease development. We consider the following two aspects in our inference approach:

(1) If a biological process is causing cancer to develop, in some point during its progression there was a critical event that cause significant disturbances deviating such a process from normal to carcinogenic. Although, in general a biological process is not disruptive, we hypothesize there is a point along the process where transition from normal to cancer becomes irreversible.
(2) If a gene is driving a biological transition relevant for cancer to develop, it should cause significant changes that can be detected on its own *pseudotime* ordered gene expression (i.e. the *pseudotime* series of the gene expression of the gene, Section 2.3.1). We use abnormal gene expression as an indicator of alterations in the behaviour of genes since the analysis of genes differentially expressed as indicators of cancer is a common and widely accepted approach.

Dynamic information required for our causal inference of drivers will depend on the process selected for study. Most of cancer datasets contain cross-sectional data, and thus dynamic information is not explicitly available. We use a covariate encoding significant aspects of the trajectory of interest, and *pseudotime* concept to extract the underlying dynamics of such a process from cross-sectional datasets. We use the framework *PhenoPath* provided by Campbell [15, 21] for scoring each sample to reflect its relative position along the biological process (or trajectory). We selected *PhenoPath* framework for *pseudotime* scoring because of its confirmed ability for extracting dynamical information from both single-cell and bulk data [15].

Our method uses *pseudotime score* to order the samples. We denominate as the *pseudotime series* of a gene $g$ to the ordered gene expression in which an observation $g_t$ denotes the observation at sample $t$ and $t \in \{1,2,...,n\}$ indexes the samples in *pseudotime* order (further discussion in Section 2.3). Our method can be summarized in three phases:

(1) Network-based selection of putative drivers (Section 2.2): A specific *Protein–Protein Interaction* (PPI) network is created from dataset. Most influential genes in this network are labelled as putative cancer drivers.
(2) Pseudotime series modelling and event detection (Section 2.3): A provided trajectory/path covariate and PPI genes in dataset are used for *pseudotime* scoring. We use the term *pseudotime* order to refer to the order of the samples that are sorted in ascending order of the score. Scoring is performed by using the *PhenoPath pseudotime* model proposed in [21]. We used the calculated *pseudotime* score for creating *pseudotime series* of both, genes and the path covariate. We evaluate each sample following *pseudotime* order to detect the ‘event’.
(3) Cancer drivers identification based of *Causal Impact* (Section 2.4): We assess how significant the changes on *pseudotime series* (after the ‘event’) are. Causal relationship between genes and the selected process in cancer (encoded by the path covariate) is assessed following a procedure inspired in the *Causal Impact* approach proposed by Brodersen et al. [25].

### 2.2 Phase 1: Network-based selection of putative driver genes

**Figure 2:**
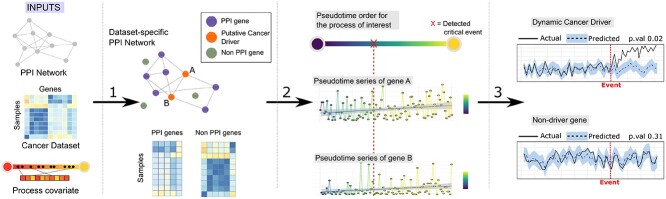
Summary of the proposed method for identifying genes driving relevant processes making cancer to develop. (1) Starting with a gene expression dataset, our method infers a dataset-specific Protein–Protein Interaction (PPI) network. Dataset is divided in PPI and Non PPI genes. Genes with the most interactions in the specific PPI network are considered putative drivers. (2) PPI genes and a trajectory covariate are used for inferring a pseudotime order that follows the process of interest encoded by the path covariate. Ordered samples are assessed to determine the most significant change along the process, or critical event. All Gene expressions in the dataset are ordered to create pseudotime series. (3) Pseudotime series of genes are used for creating a counterfactual model. *Causal Impact* is calculated as the differences between counterfactual and observed gene expression. If a significant causal effect is detected, the gene is label as ***dynamic cancer driver***. If predicted and actual gene expression values are not significantly different, the gene is labelled as non cancer driver.

Starting with a matrix $\textbf{G} \in \mathcal{R}^{n \times m}$ containing the gene expression from $n$ samples and $m$ genes, our method performs a dimensional reduction $\textbf{G}^{n \times m} \to \textbf{G}^{n \times k}$, with $k \le m$ by removing any gene not expressed in at least 20% of the samples. We use the directed protein–protein interaction (PPI) network provided by [26] as input to classify the remaining genes as ‘PPI genes’ or ‘non PPI genes’. This network consist on 34 814 gene–gene interactions from 6339 different genes. A data specific PPI network is created from $\textbf{G}$ as the PPI sub-network formed by the genes present in the dimension reduced dataset (see Figure 2, item 1).

A gene is considered a *putative driver* for our method if such a gene: (i) belongs to the data-specific PPI network inferred and (ii) the degree of its corresponding node (degree defined as the total of interactions of the gene in the inferred PPI network) is part of the top 40% of the degree of nodes distribution. Although this parameter can be adapted, our experiments show a suitable threshold stays between top 50% and top 25%.

### 2.3 Phase 2: Pseudotime series modelling and event detection

#### 2.3.1 Pseudotime series

As we have assumed cancer progression is a compendium of heterogeneous processes, there are several relevant trajectories that can be employed for our dynamic causal inference of cancer drivers. The definition of a process of interest is fulfilled by using a provided trajectory covariate $P$. It is assumed that the provided covariate $P$ encodes significant aspects of the latent progression of the samples (e.g. patients, tumours, cells, etc). Assumption of existence of such a covariate is reasonable as *pseudotime* order usually reflects progression associated with prior factors of interest [15]. In general, a numeric vector (e.g. gene expression of a biomarker) or categorical values (such as oestrogen-receptor (ER) status in breast cancer) can be used as a suitable covariate.

Formally, given an expression matrix of all ‘PPI genes’ in the dataset-specific PPI network (obtained in phase 1, see Figure 2) and a path covariate $P$, our method utilizes *pseudotime* concept to score each sample to reflect its relative position along such a process. In this paper, $P$ is considered to be a numerical (continuous) covariate. In order to be valid, selected covariate must have a unique value matching each sample in the dataset. If no *pseudotime* order is provided, our method relies on *PhenoPath* [21] for scoring each sample. The dataset samples are reordered in *pseudotime* order. Consequently, each column in the dataset now is considered a *pseudotime series* (Figure 2, item 2).

#### 2.3.2 Event detection

To detect the critical event (or event for short), we test the *pseudotime series* of the path covariate to determine the sample at which the most significant change happens (i.e. the ‘event’). Our method orders the path covariate $P$ by using the *pseudotime* score (as explained in Section 2.3.1) to create its *pseudotime series*. It is assumed the ‘event’ is not in the first five or last five samples (in *pseudotime* order). This restriction is made based on the assumption that the provided dataset contains samples reasonably dispersed along the trajectory. This requirement may not be fulfilled when using a dataset including only samples on a very limited region of the transition, and thus not reflecting adequately the progression of the biological process.

For the event detection, we used a modified version of *Causal Impact* [25] in our approach. To assess whether changes in a response variable are due to a treatment *Causal Impact* requires a time series of the response variable and a contemporaneous time series that is unaffected by the treatment. *Causal Impact* uses the unaffected time series as reference level for counterfactual estimations.

In our approach, we utilize the path covariate P as a proxy of a response variable as we expect that its pseudotime series encodes the changes due to cancer of a biological process of interest. Changes in a dynamic cancer driver are expected to be similar to the changes encoded by the path covariate. For such a reason, and because our putative drivers belong to the PPI network, we have ruled out ‘PPI genes’ as suitable references for the counterfactual estimations required by *Causal Impact*, and we only use ‘non PPI’ genes as the references. The ‘event’ identification is performed as follows:

**(a)** Find the ‘non PPI’ gene with the largest *Pearson* correlation with the path covariate $P$.
**(b)** Starting from the 5th sample (in *pseudotime* order), set current sample as ‘plausible event’.
**(c)** Use data from all samples before current ‘plausible event’ (i.e. from sample 1 to current ‘plausible event’) to fit a regression model as shown in equation 1  
  (1) \begin{align*}& \hat{P}_t = \mu_t + \delta_t + \beta \hat{x}_t+\varepsilon_t \;\;\; \text{ with} \;\;\; \varepsilon_t \sim \mathcal{N}(0,\sigma_t^2), \end{align*}
  where $\hat{P}_t$ is the predicted value of $P$ at *pseudotime* $t$, and $\hat{x}_t$ is the value at $t$ of the *pseudotime series* of the ‘non PPI gene’ selected in step **a**, $\mu _t$ is the current local linear trend level and $\delta _t$ its respective slope, $\beta $ is a regression coefficient, and $\varepsilon _t$ is the error term.
**(d)** Use model obtained in **c** to get predicted values of $P$ for all samples from current ‘plausible event’ on.
**(e)** Check whether differences between predicted and observed values are significant, and assess if those differences are due to cancer. This is done by retrieving the *Relative Causal Impact* for each ‘plausible event’, calculated as shown in equation 2 (a detailed explanation of *Causal Impact* in our method is presented in 2.4).
  (2) \begin{align*}& \text{Relative Causal Impact} = \frac{\sum_{\text{post-period}}{P_t}}{\sum_{\text{post-period}}{\hat{P}_t}} - 1 , \end{align*}
  where post-period refers to *pseudotime* values from current plausible event on, $P_t$ is actual value of $P$ at *pseudotime* $t$, and $\hat{P}_t$ is the contemporaneous predicted value.
**(f)** Label the ‘plausible event’ with the largest (significant) *Relative Causal Impact* as the identified ‘event’.

### 2.4 Phase 3: Cancer drivers identification based on Causal Impact

Given a causal relationship of two variables A $\rightarrow $ B (read as A causes B) ongoing from an arbitrary time $t_e$, the *Causal Impact* of B (the response variable) due to A (the cause) is described as the potential change (after time $t_e$) of B induced by A. In other words, the *Causal Impact* on B is the difference between observed values of B after $t_e$ and the value that B would have had if A would have not existed [25].

In our approach it is assumed that the causal effect of a driver in the biological process is reflected on the *pseudotime series* of such a driver. Consequently, we defined the *Causal Impact* of a driver gene as the difference between values due to cancer of the (observed) gene expression of such a gene, and the (unobserved) gene expression of the same gene if cancer would have not occurred. In our setting, we define $t_e$ as the index of the sample (in *pseudotime* order) where the most critical change occurred (i.e. the ‘event’). Furthermore, response variables in our approach are *pseudotime series* of putative drivers (Section 2.2). Identification of the ‘event’ $t_e$ is made as explained in Section 2.3.2.

In order to obtain the unobserved gene expression of a putative driver a counterfactual model of the respective *pseudotime series* is required. Such a counterfactual model can be achieved by using another *pseudotime series* as contemporaneous covariate to tune a regression model [27]. We select as covariate for a putative driver the ‘non PPI gene’ with the largest *Pearson* correlation with such a putative driver. We select covariates from the group of ‘non PPI genes’ as they are outside the PPI network and we hypothesize that changes in ‘PPI genes’ after the ‘event’ do not induce changes in the covariates.

The process for obtaining counterfactual estimations of a putative cancer driver is as follows: (i) determine the sample representing the most significant change along the path of interest, or ‘event’ (as shown in Section 2.3.2), (ii) find a counterfactual model of the putative driver using its data before the ‘event’ (Section 2.4.1) and (iii) use the model from (ii) to obtain counterfactual data after the ‘event’.

#### 2.4.1 Counterfactual Model

A state space representation of time series data is used as counterfactual model to estimate *pseudotime series* of putative drivers if cancer were not happened. State space representation is a widely accepted approach for modelling time series and dynamic systems. It relates observed values of variables in a system with latent states of such variables (please see [28] for a comprehensive explanation).

In this paper, given the expression profile of a putative driver gene $g \in \textbf{G}$, its corresponding *pseudotime series* is modelled using the state space model described in equations 3 and 4. Data before the ‘event’ (in *pseudotime order*) are used to fit the state space model. The model is used for obtaining counterfactual estimations (from the ‘event’ on). Specifically, we used the adapted version of [29] state representation model [25, 29, 30] as shown below:

(3) \begin{align*} {g}_t = Z_t^T\alpha_t +\varepsilon_t \;\;\; \text{ with} \;\;\; \varepsilon_t \sim \mathcal{N}(0,\sigma_t^2), \end{align*}

 

(4) \begin{align*} \alpha_{t+1}=T_t\alpha_t+R_t\eta_t \;\;\; \text{ with} \;\;\; \eta_t \sim \mathcal{N}(0,Q_t), \end{align*}

where equation 3 is known as the *observation* equation and it relates $g_t$ (an observed value of the *pseudotime series* of gene $g$) with the vector of latent states $\alpha _t = [\mu _t \;\; \delta _t \;\; 1]^T$ through the *output vector*  $Z_t^T = [1 \;\;1\;\; \beta x_t]$.

In this model, $\mu _t$ is the current local linear trend level and $\delta _t$ its respective slope, $x_t$ (used as covariate) is the contemporary value of the ‘non PPI gene’ with the largest *Pearson* correlation with $g$, $\beta $ is a regression coefficient and the $\varepsilon _t$ term is an scalar observation error following a normal distribution $\mathcal{N}(0,\sigma _t^2)$. Equation 4 is the *transition* equation and it describes the evolution of the latent states ($\alpha _t$) over time. Here, $T_t$ is a squared transition matrix, and $R_t\eta _t$ is the error term.

Counterfactual estimations $\hat{g}_t$ are achieved by evaluating equations 3 and 4 for all $t \ge t_e$ (from the ‘event’ on). These estimations are used to calculate the *Causal Impact* reflected in the *pseudotime series* of the putative driver (equation 5).

(5) $$\begin{array}{rl} & {\text{Causal\_Impact}}_{t}:=g_{t}-{\hat{g}}_{t}\;\;\;\;\;\;\forall \;\;\;t_{e}\leq t\leq n \end{array}$$

Counterfactual modelling in our method and evaluation of the significance of the *Causal Impact* are performed by using the *CausalImpact* function from the *Causal Impact* R package [25].

#### 2.4.2 Dynamic cancer drivers discovery

In this step, putative drivers with a significant *Causal Impact* detected from its own *pseudotime* series are considered ***dynamic cancer drivers*** (Figure 2, item 3). We consider there is a significant *Causal Impact* of a putative driver if the causal effect calculated by using observed and predicted gene expression values after $t_e$ (the ‘event’) is statistically significant (*CausalImpact P*-value $< 0.05$).

## 3 Results

### 3.1 Dynamic cancer drivers identified from single-cell data

We applied our method to the single-cell RNA sequencing data from NCBI GEO database, accession GSE75688 [22]. This dataset contains gene expression from 11 breast cancer patients (515 single-cell sequencing data) with distinct molecular subtypes: oestrogen receptor positive (ER+), double positive (ER+ and HER2+), human epidermal growth factor receptor 2 positive (HER2+) and triple-negative breast cancer (TNBC). We performed our inference by using 317 out of 515 cells from this dataset, corresponding to all tumour cells provided. The dataset after removing low expressed genes has the gene expression of 9551 genes.

Samples were ordered following two different *pseudotimes* calculated by using HER2 and VIM expressions as path covariates, respectively. HER2 was employed as trajectory covariate for generic breast cancer progression as it is a well-recognized oncogene whose disregulation influences breast cancer development. Similarly, VIM was used as trajectory covariate of the *Epithelial to Mesenchymal* (*EMT*) process because of the fact that VIM is a well-recognized *EMT marker*. As a result, our method identified two sets of drivers, one from each pseudotime order. For simplicity, we refer these pseudotime orders as ‘HER2time(SC)’ and ‘VIMtime(SC)’ from now on.

A total of 604 genes were inferred as drivers during generic breast cancer progression (as encoded by ‘HER2time(SC)’), while a total of 545 genes were inferred to drive *EMT* following ‘VIMtime(SC)’ from this dataset (please refer to Table 1). Our method is able to identify 98 and 93 experimentally confirmed cancer drivers listed by the *Cancer Gene Census* (v94, n = 719) [24] (also called CGC genes) from ‘HER2time(SC)’ and ‘VIMtime(SC)’, respectively. For further analysis, we combined our discoveries in one single list, resulting in 934 inferred genes. Among them, a total of 155 CGC genes were identified (Table 1).

**Table 1:** Summary of Inferred Drivers: Size of the different sets of drivers inferred from each pseudotime. Combined shows number of the union set

	HER2time(SC)	VIMtime(SC)	Combined(SC)
Total inferred	604	545	934
CGC genes	98	93	155
% of CGC genes	16.225	17.064	16.595

**Figure 3:**
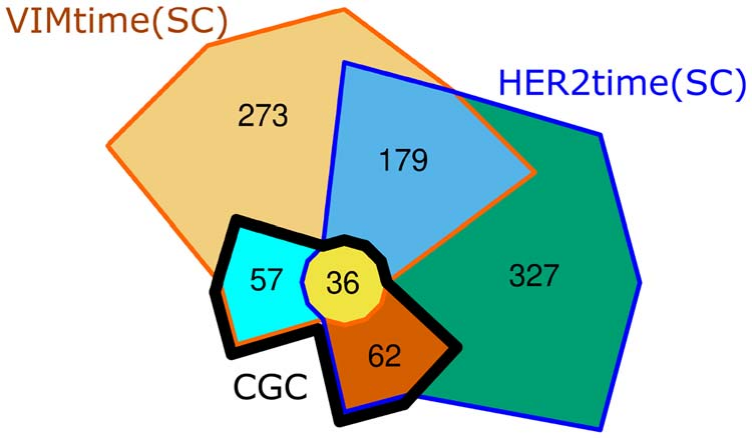
Chow–Ruskey Venn diagram of our discoveries when exploring drivers following VIMtime(SC) and HER2time(SC) order. The 155 CGC genes detected by our method correspond to 57 discovered from VIMtime(SC), 62 discovered from HER2time(SC) and 36 detected in both trajectories. Novel inferred drivers are distributed as follows: 273 inferred by following ‘VIMtime(SC)’, 327 inferred by following ‘HER2time(SC)’ and 179 genes inferred to drive both trajectories. Figure made using intervene [31] (https://intervene.shinyapps.io/intervene/).

As expected, different set of genes are identified as drivers of each biological transition of interest from our experiments. The 545 ***dynamic cancer drivers*** inferred following *EMT* as encoded by ‘VIMtime(SC)’ and the 604 inferred from ‘HER2time(SC)’ are distributed as follows: 36 CGC genes detected as drivers in both trajectories, 57 CGC genes detected by following (exclusively) ‘VIMtime(SC)’, 62 CGC genes driving cancer progression following (exclusively) ‘HER2time(SC)’, 179 non-CGC genes detected in both trajectories, 273 non-CGC genes detected from ‘VIMtime(SC)’ and 327 non-CGC genes detected from ‘HER2time(SC)’ (please see Figure 3). *Supplementary table 1* and *Supplementary table 2* (in *supplementary material*) show the top 100 ***dynamic cancer drivers*** from ‘HER2time(SC)’ and ‘VIMtime(SC)’, ranked by Relative Causal Impact (descending order), respectively. The full list of drivers inferred from the single-cell data set employed can be found at https://github.com/AndresMCB/DynamicCancerDriver.

### 3.2 Our method contributes to expanding current cancer drivers catalogues

We adapted the metric described in Dietlein et al. [2] to assess the ability of our method to contribute to the expansion of current cancer drivers catalogues. We examined the 155 CGC genes discovered as drivers from our method (union set of ‘VIMtime(SC)’ and ‘HER2time(SC)’ inferences) and compared them with CGC genes reported in previous (comprehensive) studies for driver-gene discovery. Under this approach, the ability of our method to expand previous catalogues is correlated with the number of additional CGC genes the method discovered. Additional CGC genes are genes in CGC that are inferred by our method but not present in any of the catalogues. A brief description of studies selected for this assessment is shown below.

We selected six comprehensive studies providing catalogues of highly confident drivers. Lawrence et al. provide a catalogue of driver genes obtained from 4742 tumour data by using the MutSigCV suite [32]. Martincorena et al. [33] used the dNdScv algorithm on 7664 tumours to assemble a cancer drivers list. Bailey et al. [4] create a catalogue by applying a combination of 26 different computational tools for driver genes inference to 9423 tumours. Priestley et al. [34] provide a driver mutation catalogue discovered from 2520 paired tumour and normal genomes from 2399 patients. Dietlein et al. [2] identified drivers from 11 873 tumour-normal pairs by using a novel method (MutPanning) based on nucleotide context. Finally, Rheinbay et al. [35] provide a catalogue of coding and non-coding cancer drivers inferred by analysing 2658 genomes from the Pan-Cancer Analysis of Whole Genomes (PCAWG) Consortium of the International Cancer Genome Consortium (ICGC) and The Cancer Genome Atlas (TCGA) [36].

**Figure 4:**
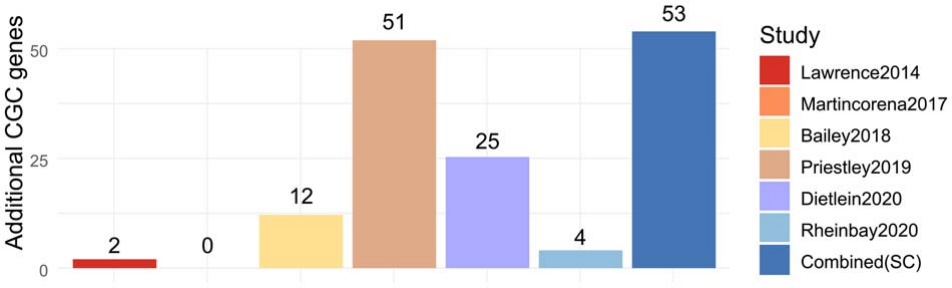
Comparative of number of novel CGC genes inferred by our method and novel CGC of each of the cancer drivers catalogues analysed. A CGC gene is considered novel for our method if such a gene does not belong to any of the analysed catalogues. Similarly, a CGC gene in a catalogue is considered novel is such a gene is not present in any other of the catalogues. Our method is capable to detect 53 novel CGC, suggesting our method is useful for expanding current comprehensive catalogues.

From the 155 CGC genes identified by our method, 53 are not part of any of the aforementioned catalogues. Similarly, we count the number of additional CGC genes that can be found in each catalogue (i.e CGC genes that are not in any other of the studies). The number of additional CGC genes from each catalogue are as follows: [34] 51; [2] 25, [4] 12, [35] 4, [32] 2 and [33] 0 (please refer to Figure 4). The above suggests that the ability of our method to detect additional drivers is useful for expanding previous studies findings.

#### 3.2.1 Stratification of inferred driver genes based on literature support

To further explore the idea of contributing to the expansion of current cancer drivers catalogues, we have assembled a stratified list of ***dynamic cancer drivers***. In our proposed catalogue we only consider the 215 genes discovered as drivers in the intersection of both *pseudotime* orders (179 novel genes and 36 CGC genes, see Figure 3) as they are relevant to both of the trajectories analysed.

Stratification corresponds to level of confidence as driver gene. We have classified the 215 inferred genes in the following four categories: **A**—the gene is a CGC gene, **B**—the gene has literature support as influential for breast cancer, **C**—the gene have literature support as influential for other cancer but breast cancer and **D**—there is not literature describing the gene as influential for cancer. Inclusion criteria for the literature review were: (i) paper explicitly refers the gene has a significant role in cancer, (ii) publication dated 2016 onwards and (iii) study is published in a journal with impact factor 5.1 or higher (as shown in *web of knowledge*, website visited in August 2021).

Among the 215 classified genes, 36 are CGC genes (A), 57 genes have been related to breast cancer (B), 69 genes have literature support regarding other cancer (C) and 53 genes have no literature support yet. For example, the receptor tyrosine kinase AXL, inferred for our method as the gene with the largest *Relative Causal Impact*, has been found to stimulate the phosphorylation of a network of focal adhesion (FA) proteins, promoting metastasis in breast cancer cells [37]. The same gene has been considered as a potential target for cancer therapy since its knockdown is related to slower growth and increased radiosensitivity in breast cancer tumours [38]. We use the *Relative Causal Impact* for ranking our discoveries as it is the percentual version of *Causal Impact*, and thus eliminates the bias towards highly expressed genes.

Genes in the top 10 of inferred drivers (ranked by *Relative Causal Impact*) are distributed as follows: one gene has been found to be a confirmed cancer driver (IRF4 ranked in top two), six genes have support from experimental studies (AXL, IGF1R, STC2, MBD3, SATB1, ENO2) and three genes has no literature support yet. IGF1R (our top five) has been found to co-regulate tumourigenes and cancer progression in breast cancer [39]. STC2 (top three) promotes *EMT* progression in colorectal cancer, inducing tumourigenesis [40]. MBD3 (top four) plays a significant role in *EMT* process and tumour metastasis [41]. SATB1 (top seven) plays a critical role in relevant pathways conferring anti-tumour immunity [42]. Lastly, knockdown of ENO2 (top nine) has been associated with a reduction on the survival rate of leukaemia cells [43]. The full stratified list can be found in *Supplementary table 3*. The full list with references from our literature review can be found in the *Supplementary* folder at https://github.com/AndresMCB/DynamicCancerDriver.

### 3.3 Dynamic cancer drivers inferred from single-cell data are significantly related to cancer disease

We performed an enrichment analysis (GO biological processes 2021) to verify how significantly related are our discoveries to processes in cancer. Additionally, we explored gene–disease associations (GDAs) among our inferred drivers by checking the overlap between our inferred genes and the human GDAs provided by DisGeNET [44].

Our analyses show a significant number of discovered drivers are strongly related to cancer disease. The top five GO Biological Processes and the top five GDAs enriched terms for VIMtime(SC) (ranked by *P*-value) are shown in Table 2. The top 10 enriched terms for each of our enrichment analyses are provided in *Supplementary table 4* (GO terms HER2time(SC)), *Supplementary table 5* (GO terms VIMtime(SC)), *Supplementary table 6* (GDAs HER2Time(SC)) and *Supplementary table 7* (GDAs VIMtime(SC)). An extended visual representation (top 10) for both GO terms and GDAs terms can be found in *supplementary material (Figures 1, 2)*. The full outcome of our analyses (including the list of enriched genes) can be found at https://github.com/AndresMCB/DynamicCancerDriver. Drivers discovered by following *EMT* process are in general related to generic processes in cancer (e.g cancinogenesis in top one and neoplasm metastasis in top five of DGAs), while drivers inferred following ‘HER2time(SC)’ are more closely related to breast cancer.

**Table 2:** Top five of GO Biological Processes (left) and DisGeNET DGAs (right) enriched terms among drivers inferred from VIMtime(SC)

**GO Biological Process Term**	**P-value**	**Disease–Gene Association Term (DisGeNET)**	***P*-value**
Positive regulation of transcription, DNA-templated (GO:0045893)	6.05E-41	Carcinogenesis	1.39E-55
Regulation of transcription by RNA polymerase II (GO:0006357)	2.28E-29	Malignant neoplasm of breast	1.64E-54
Positive regulation of transcription by RNA polymerase II (GO:0045944)	4.79E-29	Breast Carcinoma	2.31E-53
Negative regulation of transcription, DNA-templated (GO:0045892)	6.06E-29	Malignant neoplasm of prostate	3.05E-52
Regulation of transcription, DNA-templated (GO:0006355)	1.60E-27	Neoplasm Metastasis	3.88E-52

The overlap of our inferred drivers and the top three GO Biological Terms (2021) resulting from enrichment analysis are as follows: for drivers inferred by using ‘VIMtime(SC)’, positive regulation of transcription, DNA-templated (GO:0045893) (125/545, *P*-value 6.05E-41), regulation of transcription by RNA polymerase II (GO:0006357) (154/545, *P*-value 2.28E-29) and positive regulation of transcription by RNA polymerase II (GO:0045944) (93/545, 4.79E-29). For drivers discovered by following ‘HER2time(SC)’, positive regulation of transcription, DNA-templated (GO:0045893) (144/604, *P*-value 2.06E-49), positive regulation of transcription by RNA polymerase II (GO:0045944) (114/604, *P*-value 6.47E-40) and regulation of transcription by RNA polymerase II (GO:0006357) (173/604, *P*-value 1.01E-33).

Similarly, overlap between our inferred set of drivers and DisGeNET cancer associated genes includes a significant number of genes. Overlaps of our inferred drivers and the top three GDAs terms resulting from DisGeNET analysis are as follows: for drivers inferred by using ‘VIMtime(SC)’, cancinogenes (273/545, *P*-value 1.39E-55), malignant neoplasm of breast (306/545, *P*-value 1.64E-54) and breast carcinoma (301/545, *P*-value 2.31E-53). For drivers discovered by following ‘HER2time(SC)’, mammary neoplasm (244/604, *P*-value 1.71E-73), malignant neoplasm of breast (353/604, *P*-value 4.62E-69) and breast carcinoma (348/604, *P*-value 4.75E-68). These results suggest our inferred driver genes are significantly related to cancer.

### 3.4 Dynamic information enhance drivers discovery from bulk data

Cancer data from cross-sectional data, also known as bulk data, account for the current majority of data from cancer patients. For such a reason, significant coordinated efforts have been made to create repositories hosting bulk data useful for research purposes. As an example, *the cancer genome atlas (TCGA)* program (https://www.cancer.gov/tcga) provides a comprehensive collection of genomic, epigenomic, transcriptomic and proteomic cancer data [23]. Availability of these datasets is one of the reasons most of the current methods for driver discovery are designed for bulk data.

To further assess the performance of our method, and validate whether including dynamic causal inference procedures enhance the ability to identify driver genes from bulk data, we applied our method to the TCGA–BRCA cancer dataset. Data used for this analysis were downloaded using the *TCGAbiolinks* package [45]. A total of 752 primary solid tumours samples from 747 patients were selected, corresponding to the same patients analysed by Pham et al. [8]. Data downloaded consist of gene expression (raw counts) harmonized against GRCh38 (hg38). The dataset after removing low expressed genes has 19 351 genes. To allow a fair comparison with mutation-based approaches, we ranked our discoveries by frequency of single nucleotide variations in this dataset (the more times the gene appears as mutated, the higher the rank). Mutational information was obtained from Mutation Annotation Format (MAF) (MAF.hg38) by using *TCGAbiolinks*.

We normalized this dataset in order to compensate within samples and cross-sectional samples effects. This normalization was performed by using the *TCGAanalyze_Normalization* method from *TCGAbiolinks* package, which uses procedures to adjust for GC-content effect on read counts [46]. VIM and HER2 gene expression after normalization are used as path covariates to find the corresponding ‘VIMtime(Bulk)’ and ‘HER2time(Bulk)’ *pseudotime* orders from this dataset. We used the COSMIC CGC (v94, n=719) as the reference set of CCG genes.

**Figure 5:**
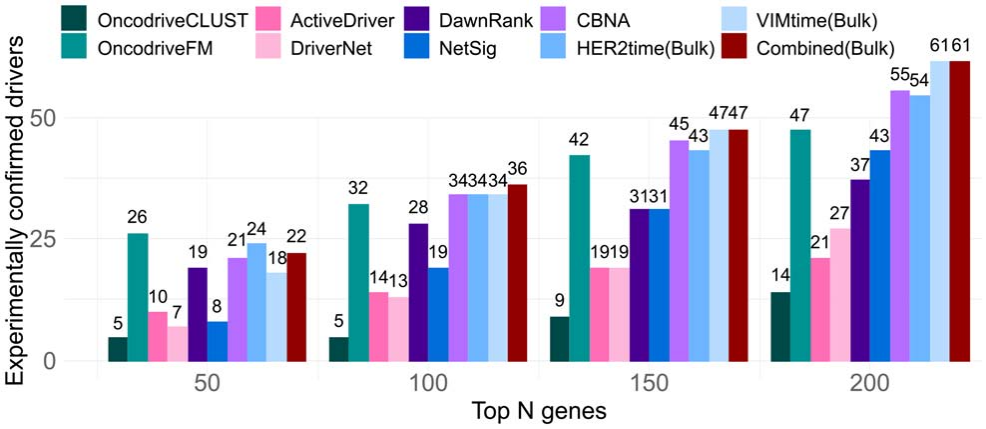
Comparison of different cancer drivers inference methods for bulk data. We benchmarked the performance of our method against seven other methods for cancer driver discovery. We use CGC genes as a conservative approximation of the ground truth as the full set of cancer driver genes remains still unknown. We present the results for top N =50,100,150,200 genes following each method’s rank. For a fair comparison with other mutational approaches, we ranked our inferred genes following mutation frequency in TCGA–BRCA dataset.

Performance of a method is assessed based on the total of CGC genes such a method is able to identify. The more CGC genes the method recall, the better the method is. To facilitate the comparison, we explore the performance of the benchmarked methods in the top 50, 100, 150 and 200 drivers predicted by each of them. OncodriveCLUST, OncodriveFM and NetSig utilize corrected *P*-value to rank their discoveries. ActiveDriver and DriverNet inferred genes are ranked by *P*-value. DawnRank provides its own ranking scores. CBNA also provides its own ranking based on the mutation frequency of genes. Results of performance are summarized in Figure 5.

The last three bars of each of the analysed top 50, 100, 150 and 200 drivers shown in Figure 5 correspond to the performance of our method for drivers inferred from ‘HER2time(Bulk)’, ‘VIMtime(Bulk)’ and combined (union set), respectively. In general, our method outperforms other methods when inference was made following *EMT* process (VIMtime) and combined (union of the two inferred sets). Inference performance following ‘HER2time’ of our method is comparable with CBNA, and outperforms other methods. These results suggest our method is able to identify mutated cancer drivers even when mutational information is not used during the inference process. Furthermore, these results also suggest that including information of the underlying dynamics (as well as causality) can enhance the ability for cancer drivers discovery, even in bulk data. The top 100 ***dynamic cancer drivers*** inferred from ‘HER2time(Bulk)’, and the top 100 from ‘VIMtime(Bulk)’ are shown in *supplementary material* (*Supplementary table 8* and *Supplementary table 9*). The full list of ***dynamic cancer drivers*** discovered in our experiments form bulk data can be found in the *Supplementary* folder at https://github.com/AndresMCB/DynamicCancerDriver.

### 3.5 Gene regulatory networks for dynamic cancer driver genes

We explored possible regulatory relationships among our dynamic cancer drivers and the GCG genes that are marked as breast cancer drivers by the COSMIC project. We retrieve regulatory relationships from the top 200 inferred DCD by using HER2 as path covariate (for both single-cell and bulk data), and the genes detected by our method that are marked as breast cancer driver in COSMIC Cancer Gene Census database [24]. Regulatory relationships are extracted from the website http://www.grndb.com [47]. We have only used the regulatory relationships from Breast Invasive Carcinoma (BRCA–TCGA) data and kept only the relationships with high confidence. The result contains 93 699 regulatory relationships. From this result we extract all relationships between the top 200 of DCDs and the set (not restricted to the top 200 DCDs) of CGC Breast Cancer drivers detected by our method (when using ‘HER2time(SC)’ and ‘HER2time(Bulk)’, respectively).

**Figure 6:**
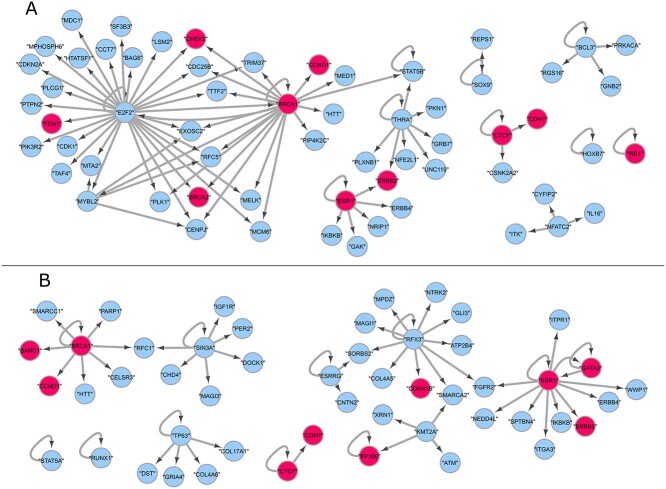
Regulatory relationships among DCDs. Gene Regulatory Networks showing genes in the union set of top 200 DCDs (blue) and the total of CGG breast cancer drivers identified as DCD (magenta). Network analysis reveals novel drivers that are highly influential in the network such as E2F2 and THRA from single cell (Figure 6 A), and SIN3A from bulk data (Figure 6 B).

Following this procedure, a total of 81 regulatory relationships were detected among the DCD genes discovered from single-cell data. Similarly, a total of 56 regulatory relationships between DCD genes were detected from bulk data. In both cases, broadly accepted breast cancer drivers such as BRCA1 and ESR1 are central nodes in the network (Figure 6).The network analysis has revealed several influential DCDs that are not curated CGC genes yet, for example E2F2 and THRA from single-cell (Figure 6 A), and SIN3A from bulk data (Figure 6 B). E2F2 has been linked to tumourigenesis, metastasis and poor overall survival in breast cancer [48]. THRA has been found to be correlated with high breast cancer mortality [49], and SIN3A mutations in breast cancer enhances cell proliferation [50]. The list of regulatory relationships used for plotting Figure 6 can be found in *Supplementary table 10* (‘DCD(HER2_SC) Reg. relationships’), and *Supplementary table 11* (‘DCD(HER2_Bulk) Reg. relationships’).

## 4 Discussion and Conclusions

In this paper, we have presented a new dynamic causal inference method for cancer driver discovery. Our approach considers cancer drivers as genes driving one or more biological processes leading to cancer. Based on this consideration, our method hypothesize that driver genes are driving the dynamics of biological processes pathologically altered and its causal influence in cancer development is reflected on its own gene expression.

To verify this hypothesis, our method performs a data transformation to unveil the underlying dynamics linked to one (or more) trajectories of interest and verifies the possible causal link between putative drivers and the progression of the biological trajectory. Furthermore, we identify the sample that represents the most significant change along the biological transition. We use that sample to determine the *Causal Impact* of the driver. If such an impact is significant, the gene is considered a cancer driver for our method.

In our method we use a PPI network to guide a knowledge-based feature selection to define our putative drivers. However, hub genes obtained from hub identification methods and other methods for finding important genes from Gene Regulatory Networks (please check [51] for a comprehensive review of GRN methods for single cell) are also suitable putative drivers for our DCD framework. Our causal inference method carefully explores such influential genes to identify ***dynamic cancer drivers*** by assessing the causal effects of the putative drivers on cancer reflected in the pseudotime ordered gene expression of those putative drivers.

In this paper, we used HER2 and VIM to demonstrate our causal framework. However, our DCD method allows the use of other known breast cancer drivers as well, for example ESR1 (details of experiment results in supplementary material, Section 3.). This capability can be extrapolated to the known drivers of other cancer types. As a result, the DCD approach is also suitable for detecting drivers in other cancer types/subtypes when some driver genes are known.

One interesting point of our method is that it can detect drivers that are not differentially expressed. Our experiment results (see supplementary material—Section 4 for details) show that 65 inferred DCDs (in the top 100 when using HER2time(Bulk)) are not DEGs between the conditions normal/cancer, including eight CGC Breast Cancer Drivers (i.e. PIK3CA, CDH1, NCOR1, MAP2K4, AKT1, CASP8, EP300 and CTCF). These results suggest that some cancer drivers’ gene expression changes are subtle enough to prevent them from being detected by DEGs analysis, and yet significantly alter the dynamics of some biological processes, causing cancer development.

Additional experiments comparing *PhenoPath* (native), and an externally provided pseudotime (i.e. *Monocle3*, a popular method for pseudotime inference [52, 53]), have shown that our method can detect DCD genes from externally provided pseudotimes (details of the experiments can be found in *supplementary material—Section 5*). *PhenoPath* allows to orient the inferred trajectory to fit a biological process of interest by using a covariate related to such a trajectory. Due to this capability of *PhenoPath* we can infer drivers of different biological processes leading to cancer progression, while other pseudotime inference methods lack this capability. As a result, in our experiments the DCD method could identify more novel drivers from different path covariates when using *PhenoPath*, supporting our hypothesis that different biological processes have different set of driver genes.

Experiments with the data used imply the superior performance of our method. We have assessed our method’s ability to detect novel drivers that can be used for expanding current cancer drivers catalogues, identifying simultaneously experimentally confirmed drivers. Our analyses suggest that ***dynamic cancer driver*** concept is useful to expand the spectrum of current cancer drivers catalogues. Additionally, our method performs well in both single-cell and bulk data, outperforming all other analysed methods in terms of the number confirmed drivers that were predicted for each method when using bulk data. The results of our tests suggest that making use of the temporal information of biological processes significantly improves cancer driver discovery. Additionally, our experiments suggest that some cancer drivers significantly influence more than one biological trajectory along cancer progression.

**Key points**

- We hypothesize that there are multiple biological processes driving cancer and each of the processes has its own drivers.
- Our method incorporates aspects from the underlying dynamics of cancer development to a causal inference approach, allowing us to uncover causal relationship of genes driving core biological process in cancer development.
- Our experiments suggest that our approach is useful for expanding cancer drivers catalogues by including novel cancer drivers that are not detected by methods based on static data.
- Due to the dynamic nature of our causal approach, our method complements current well-recognized cancer drivers discovery methods based on static data.

## 5 Author contributions statement

A.C. and T.L. conceived the experiments, A.C., V.P. and X.L. conducted the experiments. A.C. and T.L analysed the results. A.C. and T.L. wrote the manuscript. V.P., X.L, L.L. and J.L. reviewed the manuscript.

## 6 Data and code availability

Single-cell data used in our experiments are publicly available at NCBI GEO database, under accession number GSE75688 at (https://www.ncbi.nlm.nih.gov/geo/). TCGA-BRCA dataset can be accessed at https://portal.gdc.cancer.gov/. An R data version of all datasets, and codes used in this paper (including the R-package with the implementation of our method) can be found at https://github.com/AndresMCB/DynamicCancerDriver.

## Supplementary Material

Supplementary_material_elac030

## Author Biographies

**Andres M. Cifuentes-Bernal** received his B.Sc. degree in Mechatronics from the National University of Colombia and his M.Sc. degree in Mechatronics from the Nueva Granada Military University, Colombia, in 2009 and 2015, respectively. He was a professor in the Nueva Granada Military University and he is co-inventor of three Colombian Patents. He is currently working towards the PhD degree in Mathematics and Computer Science (Bioinformatics) at University of South Australia. His research interest include bioinformatics, dynamic causal discovery and control system design.

**Vu VH Pham** received his Master degree in Information Technology from the Deakin University, Australia in 2017, and his Ph.D. degree from the School of Information Technology and Mathematical Sciences, University of South Australia (UniSA) in 2021. His research interests include causal inference methods and their applications in Bioinformatics.

**Xiaomei Li** received the M.S. degree in computer science from Fuzhou University, China, in 2011. She is currently pursuing the Ph.D. degree with the University of South Australia. Her research focuses on the development of computational methods for cancer subtype and prognosis based on bulk data and single-cell sequencing data.

**Lin Liu** received the bachelor’s and master’s degrees in electronic engineering from Xidian University, China, in 1991 and 1994, respectively, and the PhD degree in computer systems engineering from the University of South Australia, in 2006. She is currently an associated professor with the University of South Australia. Her research interests include data mining, causal inference and bioinformatics

**Jiuyong Li** (Member, IEEE) received the Ph.D. degree in computer science from Griffith University, Brisbane, Australia, in 2002. He is currently a Professor with the University of South Australia (UniSA), Mawson Lakes, SA, Australia. His research work has been supported by seven Australian Research Council Discovery projects. He has led several industrial and applied projects. His main research interests include data mining, causal discovery, privacy and fairness and bioinformatics. Li is also a member of the Australian Computer Society National Committee for Artificial Intelligence.

**Thuc Duy Le** is currently an associate Professor with the UniSA STEM unit, University of South Australia. His research interest includes causal discovery methods and their applications in bioinformatics. He is also a DECRA Fellow, for the period of 2020–2022. He has served as an Academic Editor for PLOS One, and and BMC Cancer and a reviewer for many top conferences and journals in data mining and bioinformatics.
