# Supplementary material for: Dynamic cancer drivers: a causal approach for cancer driver discovery based on bio-pathological trajectories
Source: Brief Funct Genomics. 2022 Sep 19;21(6):455–65. doi: 10.1093/bfgp/elac030 (PMC10467634; doi:10.1093/bfgp/elac030)
Supplement: Supplementary_material_elac030 [file supplementary_material_elac030.pdf]

Supplementary material: “Dynamic cancer drivers: A causal approach for cancer driver discovery based on bio-pathological trajectories”

1 GO biological processes analysis

We performed a GO biological processes enrichment analysis to the single cell RNA sequencing data from NCBI GEO database, accession GSE75688 [Chung et al., 2017] to verify how significantly related are our discoveries to processes in cancer. Our analyses show a significant number of discovered drivers are strongly related relevant biological processes in cancer disease. Top ten enriched terms (for each trajectory) from Go Biological Processes are shown in Fig. 1 . For both dynamic drivers inferred sets (i.e from VIMtime(SC), and HER2time(SC)), top 10 enriched terms (ranked by p-value) are relevant to cancer (e.g. regulation of transcription and regulation of apoptotic process).

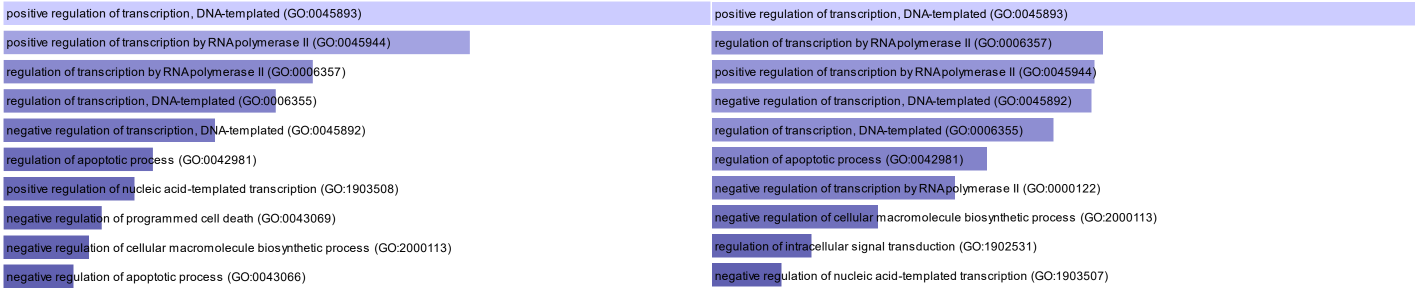

Figure 1: Top 10 Go biological terms 2021 (ranked by p-value) from our enrichment analysis for the gene set obtained from ”VIMtime(SC)” (left) and ”HER2time(SC)” (right). In both cases, enriched terms correspond to regulation of biological processes relevant to cancer progression. Bar length represents the significance of the term. Brightness is used as auxiliary visual for significance, the lower the p-value, the brighter the colour. Analysis performed by using Enrichr [Kuleshov et al., 2016]

2 Gene-disease association analysis (DisGeNet)

We performed an enrichment analysis to identify gene-disease associations (GDAs) in the inferred drivers from the GSE75688 single cell dataset [Chung et al., 2017]. We use DisGeNET [Pi˜nero et al., 2016] as gene-disease associations database for this analysis. Our analyses show a significant number of discovered drivers are strongly related to cancer disease. The top ten enriched GDAs terms (ranked by p-value) for each of the analysed pseudotimes (i.e VIMtime(SC), and HER2time(SC)) are shown in Fig. 2. In both cases, all of top ten terms are related to cancer disease and cancer progression.

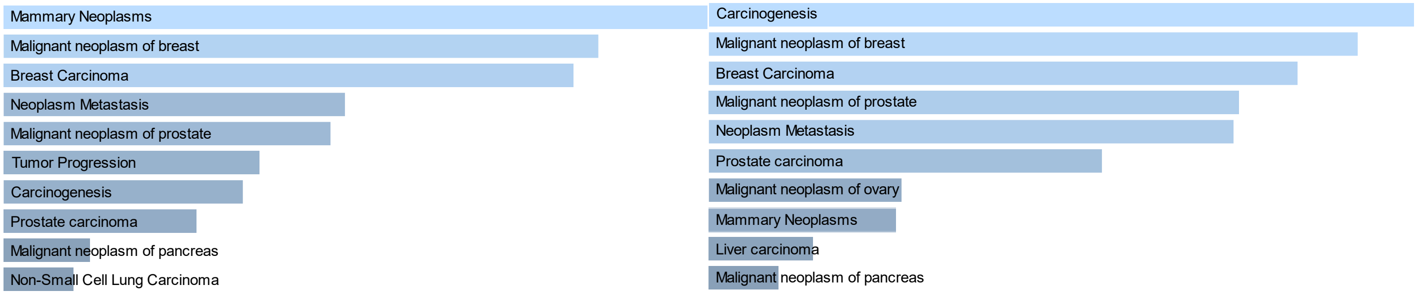

Figure 2: Top 10 GDAs terms (ranked by p-value) from our enrichment analysis for the gene set obtained from “VIMtime(SC)” (right) and ”HER2time(SC)” (left) retrieved from DisGeNET. Bar length represents the significance of the term. Brightness is used as auxiliary visual for significance, the lower the p-value, the brighter the colour. Analysis performed by using Enrichr [Kuleshov et al., 2016]

3. Additional experiments using ESR1 as path covariate:

Thanks to the capability of our method to use a gene as covariate for orienting an inferred trajectory that fits a biological process of interest, our method allows the use of well recognised cancer drivers as path covariate to potentially discover DCD for different cancer types/subtypes.

To further explore our method usability and stability when well recognised cancer drivers are used as path covariate, we have performed additional experiments by using ESR1 gene expression as path covariate. We select ESR1 since this gene is a curated breast cancer driver (CGC rank: tier 1). Our results from single cell data reveals a consistent behaviour of our method, identifying a similar number of novel dynamic cancer drivers and CGC genes when using ESR1time in comparison with our original experiments (HER2time, and VIMtime). Similarly, our additional experiments from bulk data reveals that our method outperforms most of the benchmarked methods in detecting CGC genes when using ESR1time. Results of experiments by using ESR1 as path covariate are summarised below (table 3.1, and figure 3.1). The full list of dynamic cancer drivers can be found in supplementary table 12 - dynamic cancer drivers ESR1time(SC), and supplementary table 13 - dynamic cancer drivers ESR1time(Bulk)

Table 3.1 *Summary of inferred sets. Our method displays a similar behaviour when we use a well-recognised breast cancer driver as covariate to orient the pseudotime scoring process in comparison with our original experiments.*

	ESR1time(SC)	HER2time(SC)	VIMtime(SC)
DCD inferred	500	604	545
CGC genes identified	83	98	93
% of CGC genes	16.6 %	16.255 %	17.064 %

Figure 3.1 *Comparison of different cancer drivers inference methods for bulk data. We have replicated the benchmarking process described in the original paper. Our method outperforms most methods and is the best method when including the top 200 of DCD. This result suggest that curated cancer drivers are suitable path covariates for our method.*

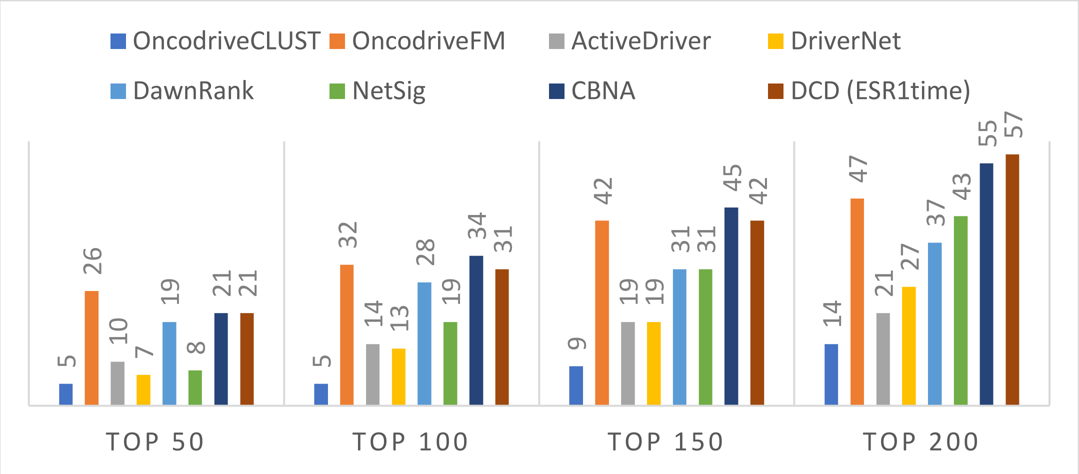

4. Differentially expressed genes (DEGs) analysis:

As an additional experiment, we performed a differentially expressed genes (DEGs) analysis to identify whether our detected dynamic cancer drivers are differentially expressed between the conditions normal and cancer. DEG analysis was performed on the TCGA-BRCA (as accessed on April 2021) by keeping all samples from normal tissue and primary tumour. This dataset has 1221 samples (113 normal samples and 1101 primary tumour samples). An R version of the dataset used can be found at http://4llab.net/Bioinformatics , in the section "Datasets".

A total of 19505 genes, after removing low expressed genes (not expressed in at least 20% of the samples) were analysed. We use the TCGAanalyze_DEA function (fdr.cut = 0.01, logFC.cut = 1, method = "glmLRT") from the package TCGAbiolinks for the analysis. A total of 6156 genes were identified as differentially expressed. The full list of DEGs can be found at supplementary table 14 ("TCGA-BRCA DEGs").

We contrasted the DEA results with the top 100 DCD inferred from "HER2time(Bulk)" and the top 100 DCD inferred from "VIMtime(Bulk)". Our experiments show that in both cases, a significant number of DCD were not detected as DEGs. Specifically, 65 DCD discovered by using "HER2time(Bulk)" and 60 DCD from "VIMtime(Bulk)" are not retrieved as differentially expressed between the conditions normal/cancer from our dataset. Above results suggest that some cancer driver's gene expression changes are subtle enough to prevent them to be detected by DEGs analysis, and yet significantly alter the dynamics of some biological processes, causing cancer development.

Finally, we filtered the CGC genes in each top 100 that are not DEGs to keep only genes that has been found to drive breast cancer. When we use "HER2time(Bulk)", our results show that 8 out of the 26 dynamic cancer drivers are curated breast cancer drivers, but they are not detected as differentially expressed in our dataset (supplementary table 15). Similarly, analysis to the top 100 DCDs when using "VIMtime(Bulk)" reveals that 7 out of the 24 CGC genes detected by our method are curated breast cancer drivers, but they are not detected as DEGs (supplementary table 16). These results support our hypothesis that the underlying dynamics of the disease provides insightful information that improves cancer driver detection. A summary of our DEA comparison is presented in table 4.1.

Table 4.1. *Comparison between DEGs and DCD from the TCGA-BRCA dataset. A DEG analysis between conditions normal tissue and primary tumour was performed to verify if our discoveries are DEGs. The top 100 DCD from HER2time and VIMtime are compared. Our results suggest that a significant number changes in gene expression between the condition normal/cancer are subtle enough to prevent to detect some drivers as DEGs.*

	not DEGs in top 100 DCD	CGC genes not DE in top 100 DCD	BRCA CD not DE in top 100 DCD
By using HER2time(Bulk)	65	26	8
By using VIMtime(Bulk)	60	24	7

5. DCD approach is useful for detecting drivers from provided pseudotimes:

Our DCD approach incorporates PhenoPath for pseudotime order scoring as it allows to orient the inferred trajectory to fit a biological process of interest by using a covariate related to such a trajectory. Thanks to this capability we can infer drivers of different biological processes leading to cancer progression. Other pseudotime inference methods lack this capability, limiting the ability of our method for detecting drivers of different altered biological processes. However, our approach has proved to be useful for detecting cancer drivers from pseudotimes provided by external methods. Please note that under these conditions, the path covariate is used only for detecting the critical event and not for orienting the trajectory to fit a biological process. As a result, the inferred DCD for different covariates (e.g. HER2, VIM) are expected to be similar.

To assess the above, we have performed further experiments by using Monocle3 (a popular method for pseudotime inference) on the single cell dataset GSE75688. For comparison purposes, we have used HER2 and VIM gene expressions as covariate to detect the critical event seen from the trajectory inferred for Monocle3. We have used the Jaccard similarity index (J.I.) to assess the similarity of the set of drivers inferred when using VIM and the set of drivers inferred when using HER2. The sets of driver genes inferred when using HER2 and when using VIM are significantly similar for the pseudotime obtained by Monocle3 (J.I. 0.7540). This result is not surprising as the method infers one "overall" trajectory from the dataset. In the other hand, the Jaccard similarity index for the sets of DCD inferred by using PhenoPath pseudotime with covariates HER2 and VIM is significantly lower (J.I. 0.2301), supporting our hypothesis that different biological processes have different set of driver genes. We have summarised these results in table 4. The full list of DCD inferred by using the Monocle3 pseudotime can be found in supplementary table

Table 4. Comparison of DCD inferred by using PhenoPath (native), and an externally provided pseudotime (i.e. Monocle3). Our method can detect DCD genes from externally provided pseudotimes. Most pseudotime methods are not able to orient the trajectory for different path covariates. As a result, our method can detect more novel drivers from different path covariates when using PhenoPath.

	Monocle3 all DCDs	*Monocle3 CGC genes*	*PhenoPath all DCDs*	*PhenoPath CGC genes*
DCD (HER2)	*687*	*120*	*604*	*98*
DCD (VIM)	*665*	*126*	*545*	*93*
Jaccard similarity index	*0.7468*	*0.7447*	*0.2301*	*0.2322*

Supplementary table 1: Top 100 dynamic cancer drivers from HER2time(SC), ranked by Relative Causal Impact (descending order).

A full comprehensive list with the 604 drivers inferred from HER2time(SC) can be found in ***Supplementary/supplementary table 1 - dynamic cancer drivers HER2time(SC).csv*** at github.com/AndresMCB/DynamicCancerDriver. Alternatively, it can be accessed at https://doi.org/10.6084/m9.figshare.20448384

	Ensembl.ID	HGNC.symbol	RelEffect	AbsEffect	p			Ensembl.ID	HGNC.symbol	RelEffect	AbsEffect	p
**1**	ENSG00000157110	RBPMS	4178.1113	18.84192	0.005		**51**	ENSG00000101096	NFATC2	16.875814	2.284409	0.005236
**2**	ENSG00000175899	A2M	1774.7859	131.9763	0.005		**52**	ENSG00000135047	CTSL	16.810349	41.42689	0.005
**3**	ENSG00000182568	SATB1	1367.9053	10.40657	0.005		**53**	ENSG00000116830	TTF2	16.223873	15.27008	0.005236
**4**	ENSG00000091831	ESR1	995.47129	51.01265	0.005		**54**	ENSG00000136936	XPA	16.13043	5.322963	0.005
**5**	ENSG00000108395	TRIM37	591.49912	12.86022	0.005		**55**	ENSG00000146232	NFKBIE	15.328779	8.028467	0.02
**6**	ENSG00000113263	ITK	550.15856	6.631933	0.005		**56**	ENSG00000104490	NCALD	15.009837	19.39583	0.005
**7**	ENSG00000163629	PTPN13	424.26875	9.785457	0.005		**57**	ENSG00000104365	IKBKB	13.722595	16.2571	0.005236
**8**	ENSG00000118785	SPP1	404.61913	62.07385	0.005		**58**	ENSG00000114739	ACVR2B	13.430946	2.026362	0.005
**9**	ENSG00000108821	COL1A1	251.6411	2.975795	0.005		**59**	ENSG00000196313	POM121	12.325685	4.433905	0.005236
**10**	ENSG00000163703	CRELD1	236.81685	10.45121	0.0052		**60**	ENSG00000105647	PIK3R2	11.368366	3.261913	0.005
**11**	ENSG00000186416	NKRF	178.36777	8.43429	0.0052		**61**	ENSG00000132664	POLR3F	11.205922	8.211849	0.01
**12**	ENSG00000109846	CRYAB	146.40606	104.3454	0.005		**62**	ENSG00000180530	NRIP1	11.199377	11.37618	0.005236
**13**	ENSG00000070061	ELP1	137.76027	8.727036	0.0052		**63**	ENSG00000205250	E2F4	11.02081	7.398753	0.005
**14**	ENSG00000197265	GTF2E2	131.08964	12.08875	0.005		**64**	ENSG00000101017	CD40	10.292378	4.942609	0.005
**15**	ENSG00000172349	IL16	125.01415	2.10021	0.005		**65**	ENSG00000196154	S100A4	10.169565	48.03548	0.005
**16**	ENSG00000131408	NR1H2	105.84597	7.703012	0.005		**66**	ENSG00000186318	BACE1	9.657818	4.380395	0.005
**17**	ENSG00000171132	PRKCE	103.07024	1.870311	0.005		**67**	ENSG00000066117	SMARCD1	9.3683661	3.478998	0.005236
**18**	ENSG00000111674	ENO2	101.79179	14.54598	0.005		**68**	ENSG00000134982	APC	9.2922105	13.05558	0.005
**19**	ENSG00000183765	CHEK2	95.229589	12.38366	0.005		**69**	ENSG00000172354	GNB2	9.2880539	7.935466	0.005236
**20**	ENSG00000148180	GSN	83.66463	14.05611	0.005		**70**	ENSG00000068028	RASSF1	9.2595008	9.923702	0.005
**21**	ENSG00000126934	MAP2K2	75.353403	5.777444	0.0052		**71**	ENSG00000133895	MEN1	8.7508287	1.59018	0.005236
**22**	ENSG00000184634	MED12	72.953482	4.791079	0.005		**72**	ENSG00000109472	CPE	8.5848494	39.94366	0.005
**23**	ENSG00000181220	ZNF746	67.588168	2.68988	0.005		**73**	ENSG00000071539	TRIP13	8.5208795	3.96534	0.005236
**24**	ENSG00000103168	TAF1C	57.452046	2.234641	0.005		**74**	ENSG00000082074	FYB1	8.4927284	2.457428	0.005155
**25**	ENSG00000107438	PDLIM1	55.015514	49.97263	0.02		**75**	ENSG00000151422	FER	8.3451181	6.523093	0.005
**26**	ENSG00000127445	PIN1	54.804242	6.064614	0.0051		**76**	ENSG00000197971	MBP	8.2837445	4.458641	0.005
**27**	ENSG00000158869	FCER1G	48.953183	17.90782	0.005		**77**	ENSG00000185043	CIB1	7.7948076	150.8311	0.015
**28**	ENSG00000149480	MTA2	47.737581	1.044046	0.005		**78**	ENSG00000178104	PDE4DIP	7.7314185	20.31789	0.005
**29**	ENSG00000197386	HTT	46.86594	21.22442	0.0052		**79**	ENSG00000135698	MPHOSPH6	7.726587	60.67041	0.005236
**30**	ENSG00000103496	STX4	46.708316	14.65071	0.015		**80**	ENSG00000272325	NUDT3	7.4441298	15.74288	0.005
**31**	ENSG00000171700	RGS19	45.073173	1.215949	0.005		**81**	ENSG00000055163	CYFIP2	7.4422544	10.84894	0.005
**32**	ENSG00000113140	SPARC	43.831426	13.14772	0.005		**82**	ENSG00000196924	FLNA	7.3470104	6.894572	0.005236
**33**	ENSG00000165304	MELK	43.044883	9.925775	0.0052		**83**	ENSG00000070770	CSNK2A2	7.201422	31.81628	0.005
**34**	ENSG00000109103	UNC119	42.229918	17.00491	0.0418		**84**	ENSG00000063244	U2AF2	7.1385251	2.994136	0.005155
**35**	ENSG00000166908	PIP4K2C	40.160629	22.37643	0.005		**85**	ENSG00000184557	SOCS3	6.9993719	2.117139	0.005
**36**	ENSG00000130713	EXOSC2	28.514143	20.76501	0.005		**86**	ENSG00000004142	POLDIP2	6.877592	23.11806	0.005236
**37**	ENSG00000071655	MBD3	28.04492	2.339941	0.005		**87**	ENSG00000123143	PKN1	6.8031887	2.901177	0.005
**38**	ENSG00000151849	CENPJ	28.011625	6.079306	0.0052		**88**	ENSG00000185651	UBE2L3	6.8006707	110.0882	0.035
**39**	ENSG00000167601	AXL	27.116727	8.841844	0.0052		**89**	ENSG00000107281	NPDC1	6.7156605	7.54119	0.005
**40**	ENSG00000150760	DOCK1	26.782633	7.742055	0.005		**90**	ENSG00000157933	SKI	6.7080208	0.898388	0.005
**41**	ENSG00000002822	MAD1L1	26.292489	6.150542	0.0052		**91**	ENSG00000104814	MAP4K1	6.6054091	7.019274	0.005236
**42**	ENSG00000165140	FBP1	25.566658	38.30417	0.0052		**92**	ENSG00000076003	MCM6	6.4645929	9.726372	0.005464
**43**	ENSG00000108854	SMURF2	24.52682	13.81009	0.025		**93**	ENSG00000138443	ABI2	6.3617729	11.09852	0.005
**44**	ENSG00000002016	RAD52	23.73388	14.02295	0.005		**94**	ENSG00000112062	MAPK14	6.2969675	15.10033	0.005236
**45**	ENSG00000149091	DGKZ	22.508349	3.367526	0.005		**95**	ENSG00000143333	RGS16	6.1774093	23.61302	0.005
**46**	ENSG00000079277	MKNK1	21.785955	16.83497	0.005		**96**	ENSG00000141644	MBD1	5.9671471	6.786761	0.005
**47**	ENSG00000182621	PLCB1	20.18339	9.031744	0.005		**97**	ENSG00000069399	BCL3	5.8257944	1.633143	0.041885
**48**	ENSG00000160294	MCM3AP	19.992472	8.580309	0.005		**98**	ENSG00000204463	BAG6	5.8257488	34.55687	0.005
**49**	ENSG00000109339	MAPK10	17.250088	17.85521	0.005		**99**	ENSG00000173020	GRK2	5.7530544	1.199343	0.005
**50**	ENSG00000101400	SNTA1	16.97895	3.241013	0.005		**100**	ENSG00000140443	IGF1R	5.6630238	29.15377	0.005

Supplementary table 2: Top 100 dynamic cancer drivers from VIMtime(SC), ranked by Relative Causal Impact (descending order).

A full comprehensive list with the 545 drivers inferred from VIMtime(SC) can be found in ***Supplementary/supplementary table 2 - dynamic cancer drivers VIMtime(SC).csv*** at github.com/AndresMCB/DynamicCancerDriver. Alternatively, it can be accessed at https://doi.org/10.6084/m9.figshare.20448384

	Ensembl.ID	HGNC.symbol	RelEffect	AbsEffect	p			Ensembl.ID	HGNC.symbol	RelEffect	AbsEffect	p
**1**	ENSG00000167601	AXL	967.0142	9.19085	0.005		**51**	ENSG00000131323	TRAF3	7.043142	2.680371	0.015
**2**	ENSG00000026025	VIM	463.5813	48.21586	0.005		**52**	ENSG00000126351	THRA	6.92421	6.414458	0.005
**3**	ENSG00000171843	MLLT3	176.5936	34.9199	0.005		**53**	ENSG00000149782	PLCB3	6.329103	0.876288	0.005236
**4**	ENSG00000137265	IRF4	147.9162	6.385924	0.005		**54**	ENSG00000091073	DTX2	6.300156	6.078588	0.005
**5**	ENSG00000118971	CCND2	126.1112	5.48083	0.0054		**55**	ENSG00000101189	MRGBP	6.244981	5.324855	0.032787
**6**	ENSG00000113739	STC2	113.5235	10.66576	0.0050		**56**	ENSG00000118785	SPP1	6.167655	53.54017	0.005464
**7**	ENSG00000057657	PRDM1	92.68285	7.910361	0.0327		**57**	ENSG00000114739	ACVR2B	6.003658	1.868845	0.005236
**8**	ENSG00000071655	MBD3	82.45974	2.402318	0.005		**58**	ENSG00000069974	RAB27A	5.893092	4.606596	0.005464
**9**	ENSG00000028137	TNFRSF1B	63.52454	2.041571	0.005		**59**	ENSG00000002822	MAD1L1	5.814716	5.441066	0.005
**10**	ENSG00000168078	PBK	63.28292	9.931831	0.005		**60**	ENSG00000154710	RABGEF1	5.579696	13.64613	0.005
**11**	ENSG00000182568	SATB1	60.5641	10.27128	0.005		**61**	ENSG00000087077	TRIP6	5.547595	5.577101	0.005236
**12**	ENSG00000010610	CD4	56.69995	5.148583	0.0054		**62**	ENSG00000129675	ARHGEF6	5.439333	5.522459	0.005464
**13**	ENSG00000111674	ENO2	55.86033	14.47186	0.005		**63**	ENSG00000097007	ABL1	5.432701	3.58441	0.005155
**14**	ENSG00000105926	PALS2	55.02897	9.489363	0.005		**64**	ENSG00000163513	TGFBR2	5.271851	2.315112	0.005464
**15**	ENSG00000117676	RPS6KA1	54.11415	9.331178	0.0054		**65**	ENSG00000157873	TNFRSF14	5.147366	3.952381	0.005076
**16**	ENSG00000132906	CASP9	50.24631	2.461116	0.005		**66**	ENSG00000108599	AKAP10	5.012705	12.19545	0.005
**17**	ENSG00000186318	BACE1	50.0033	4.759209	0.005		**67**	ENSG00000090061	CCNK	4.976074	16.32538	0.005
**18**	ENSG00000131773	KHDRBS3	38.77092	27.90944	0.0163		**68**	ENSG00000134909	ARHGAP32	4.867756	11.70782	0.005
**19**	ENSG00000141456	PELP1	37.1198	2.241423	0.005		**69**	ENSG00000182979	MTA1	4.842439	0.738495	0.005
**20**	ENSG00000077684	JADE1	36.06523	4.29893	0.005		**70**	ENSG00000168884	TNIP2	4.80785	2.693697	0.005
**21**	ENSG00000173757	STAT5B	33.83659	6.183731	0.005		**71**	ENSG00000113140	SPARC	4.789958	11.11228	0.005
**22**	ENSG00000111145	ELK3	30.53059	3.918399	0.0054		**72**	ENSG00000149311	ATM	4.789085	11.01685	0.005236
**23**	ENSG00000105647	PIK3R2	30.15169	3.444846	0.005		**73**	ENSG00000110713	NUP98	4.773384	38.32559	0.005
**24**	ENSG00000137486	ARRB1	29.05979	4.284124	0.0054		**74**	ENSG00000090104	RGS1	4.718807	71.36185	0.005
**25**	ENSG00000175104	TRAF6	28.96674	9.011639	0.0054		**75**	ENSG00000151276	MAGI1	4.455276	4.736045	0.005464
**26**	ENSG00000163703	CRELD1	24.05496	10.10235	0.005		**76**	ENSG00000104365	IKBKB	4.432541	14.23666	0.005
**27**	ENSG00000115414	FN1	23.37931	52.62427	0.0054		**77**	ENSG00000165029	ABCA1	4.128617	4.094829	0.005
**28**	ENSG00000175220	ARHGAP1	19.80497	78.5678	0.005		**78**	ENSG00000121931	LRIF1	4.083649	16.02418	0.005236
**29**	ENSG00000160972	PPP1R16A	18.59525	4.478329	0.005		**79**	ENSG00000165140	FBP1	3.915504	31.62359	0.005
**30**	ENSG00000162236	STX5	18.02857	6.38711	0.005		**80**	ENSG00000125952	MAX	3.834127	10.63444	0.038251
**31**	ENSG00000119689	DLST	16.45453	7.33199	0.0054		**81**	ENSG00000176903	PNMA1	3.726582	3.425579	0.005
**32**	ENSG00000100320	RBFOX2	16.0485	24.79369	0.005		**82**	ENSG00000143622	RIT1	3.681624	50.47222	0.005
**33**	ENSG00000003400	CASP10	15.96417	0.997455	0.0054		**83**	ENSG00000096968	JAK2	3.587398	1.709275	0.015707
**34**	ENSG00000185950	IRS2	13.8896	2.315517	0.0052		**84**	ENSG00000132485	ZRANB2	3.561511	30.98357	0.005
**35**	ENSG00000136936	XPA	13.17041	5.265493	0.0052		**85**	ENSG00000077782	FGFR1	3.528573	30.96438	0.005
**36**	ENSG00000173020	GRK2	12.7959	1.312553	0.005		**86**	ENSG00000160310	PRMT2	3.285659	29.13423	0.005236
**37**	ENSG00000120696	KBTBD7	12.36385	4.717568	0.005		**87**	ENSG00000153071	DAB2	3.205532	4.340487	0.005
**38**	ENSG00000127511	SIN3B	11.59642	2.969183	0.0163		**88**	ENSG00000077942	FBLN1	3.194051	9.569679	0.005
**39**	ENSG00000146648	EGFR	11.59405	5.714505	0.005		**89**	ENSG00000170542	SERPINB9	3.154632	29.79213	0.005
**40**	ENSG00000127946	HIP1	10.03739	7.027747	0.005		**90**	ENSG00000135966	TGFBRAP1	3.123153	3.721607	0.005
**41**	ENSG00000055163	CYFIP2	9.944083	11.21787	0.005		**91**	ENSG00000157110	RBPMS	3.07377	14.16062	0.005
**42**	ENSG00000109103	UNC119	9.843793	15.89718	0.005		**92**	ENSG00000082781	ITGB5	2.980313	18.00619	0.005
**43**	ENSG00000142166	IFNAR1	9.777363	33.20074	0.005		**93**	ENSG00000114316	USP4	2.943463	24.49412	0.005
**44**	ENSG00000103319	EEF2K	9.547039	7.269116	0.0050		**94**	ENSG00000103168	TAF1C	2.938426	1.694229	0.005
**45**	ENSG00000211899	IGHM	9.371626	5.401124	0.005		**95**	ENSG00000095794	CREM	2.900339	38.05375	0.005236
**46**	ENSG00000105221	AKT2	9.1333	2.720025	0.005		**96**	ENSG00000117318	ID3	2.728742	38.57592	0.005
**47**	ENSG00000145604	SKP2	8.58281	8.252342	0.005		**97**	ENSG00000091831	ESR1	2.728374	37.22242	0.02
**48**	ENSG00000105851	PIK3CG	8.454076	0.414084	0.005		**98**	ENSG00000178209	PLEC	2.710291	1.874952	0.005
**49**	ENSG00000020633	RUNX3	8.276306	4.934203	0.005		**99**	ENSG00000169016	E2F6	2.699985	8.172608	0.045
**50**	ENSG00000107281	NPDC1	7.478224	7.652843	0.0052		**100**	ENSG00000127824	TUBA4A	2.687856	31.44567	0.005

Supplementary table 3: Stratification of inferred driver genes (intersection set) based on literature support. Stratified list of the 215 genes discovered as drivers in the intersection of both pseudotime orders (VIMtime(SC) and Her2time(SC)). This list and the list of references analysed can be found in ***Supplementary/ supplementary table 3 - DCD stratification.xlsx*** at github.com/AndresMCB/DynamicCancerDriver. Alternatively, it can be accessed at https://doi.org/10.6084/m9.figshare.20448384

HGNC.symbol	Support in brest cancer (ref.)	Support in other cancer (ref.)	Category		HGNC.symbol	Support in brest cancer (ref.)	Support in other cancer (ref.)	Category
AXL	1,3	2,4	B		E2F6		81, 82, 83, 84, 85	C
IRF4			A		SNUPN		86	C
STC2		5,6	C		ZNF76	NA	NA	D
MBD3		7	C		MCF2L		87	C
IGF1R	8,10,11	9,12,13,14,15	B		DLG5	88	89	B
TNFRSF1B	NA	NA	D		BNIP3L		90, 91, 92, 93	C
SATB1		16,17	C		FBXW7			A
CD4	NA	NA	D		PTPRA		94, 95	C
ENO2		18	C		TAF10	NA	NA	D
RPS6KA1	NA	NA	D		MAPK10		96, 97	C
CASP9			A		S100A4	99	98, 100, 101, 102	B
BACE1	NA	NA	D		TAF6	NA	NA	D
JADE1	NA	NA	D		NRAS			A
STAT5B			A		CDK14	108	103, 104, 105, 106, 107	B
CRYAB		19, 20	C		PLK1	109, 111	110, 112, 113	B
PIK3R2	30	29	B		AKAP1		114	C
TRAF6	25,28	21,22,23,24,26,27	B		MDC1	115, 116, 118	117	B
A2M	NA	31	C		FUNDC2	NA	NA	D
CRELD1	NA	NA	D		EID1	NA	NA	D
XPA			A		ABCF3	NA	NA	D
GRK2	34,35	32,33	B		PRSS23		119	C
EGFR			A		TPD52L1		120	C
CYFIP2	NA	NA	D		IGFBP5	122, 123	121, 124	B
UNC119		36	C		SGK1			A
RUNX3	42	37,38,39,40,41,43	B		KHSRP		125, 126, 127	C
NPDC1	NA	NA	D		PSMF1		128	C
THRA	NA	NA	D		CHMP3	129		B
DTX2	NA	NA	D		BAG1		130, 131	C
SPP1	50	44,45, 46, 47, 48, 49	B		TSC1			A
ACVR2B	NA	NA	D		ELP1		132, 133, 134	C
MAD1L1		51	C		BLOC1S2	NA	NA	D
ASH2L		52,53,54	C		CTSB	135	136	B
RABGEF1	NA	55	C		EXOSC10		137	C
ABL1			A		OGT	142	138, 139, 140, 141	B
TGFBR2			A		EIF3E			A
TNFRSF14			A		CD44	143	144, 145, 146	B
AKAP10	NA	NA	D		PARD6B	NA	NA	D
SPARC	60, 62, 63	56, 57, 58, 59, 61	B		HTRA2		147, 148	C
NUP98			A		ARHGEF11	149		B
RGS1	65	64	B		EXOSC2		150	C
IKBKB			A		APP	151	152	B
LRIF1	NA	NA	D		HNRNPUL1		153, 154	C
FBP1	68, 70	66, 67, 69, 71	B		GRB7	159	155, 156, 157, 158	B
RIT1		72	C		POLR3F	NA	NA	D
FGFR1			A		CDK5RAP2	NA	NA	D
MAPT	73	74, 75	B		HOXB7	162	160, 161, 163, 164	B
SERPINB9		76, 77, 78	C		LUC7L2	NA	NA	D
RBPMS		79, 80	C		ECH1		165	C
TAF1C	NA	NA	D		UBQLN4		166, 167	C
ESR1			A		SUFU			A
ATF1			A		CRK	254, 255, 256, 257	252, 253, 253	B

DAZAP2		168	C		ARHGAP5			A
COL1A1			A		PDIA3		258	C
MDM2			A		HRAS			A
SAFB		169	C		LNX1		259, 260	C
HSF2		170, 171	C		SIN3A	261, 262, 263		B
ITSN1		172	C		REPS1	NA	NA	D
TIAM1	173, 174	175, 176, 177	B		MBIP		264	C
CDKN2A			A		SORBS2	268	265, 266, 267	B
TAF4	NA	NA	D		SNX6	NA	NA	D
HDAC3	181, 182	178, 179, 180	B		DDIT3			A
NFYA	186	183, 184, 185	B		MCM3AP	NA	NA	D
MAFG	191	187, 188, 189, 190	B		NAGK	NA	NA	D
SNW1	NA	NA	D		ZMYND8	270, 271	269, 272	B
RAB4A	193, 194	192	B		ZYX		273, 274	C
IRAK1		195, 196, 197	C		POM121		275	C
CDK9	201	198, 199, 200	B		VDR	277, 278	276	B
KRT19	203, 204	202, 205	B		SF3B3	280	279, 281	B
RHOQ	NA	NA	D		CFL1	284	282, 283	B
NFKBIA		206	C		CEBPZ	NA	NA	D
SAP30	NA	NA	D		TGS1	NA	NA	D
ZNF250	NA	NA	D		HES1	286	285, 287	B
COPS2		207, 208	C		CDH1			A
SOS1	212	209, 210, 211	B		NCL	289	288, 290	B
NRIP1		213, 214	C		ELF1		291,292	C
MED8		215	C		SNAPIN	NA	NA	D
TFE3			A		SFPQ			A
NEDD9	218	216, 217	B		MBD2	293	294, 295	B
MCM5		219,220	C		NRBP1		296	C
VASP	222, 223, 224	221	B		ELOC	NA	NA	D
SERTAD1		225	C		FUBP1			A
USP7	229, 230	226, 227, 228	B		PPHLN1	NA	NA	D
PTPRS		231	C		ERH		297, 298	C
C1QBP		232	C		EIF4G2		299, 300, 301	C
GAK		233	C		ATP5F1B	NA	NA	D
CBX4	236	234, 235	B		GOLM1	304	302, 303, 305	B
POU2F1		237, 238, 239, 240	C		ZMYND11		306	C
CCT7		241	C		XPO1			A
HCFC1		242, 243	C		ZNF165	307		B
HNRNPD		244	C		RBBP7	NA	NA	D
TUBB		245	C		TADA3	NA	NA	D
MAP2K2			A		SYNCRIP	309	308	B
EIF2AK2	248	246, 247	B		CTNNA1	312	310, 311	B
HTATSF1		249	C		RERE	NA	NA	D
STX4	NA	NA	D		EXOSC8		313	C
KDM5A			A		TAPBP	NA	NA	D
RAB2A	251	250	B		PTEN			A
CYTH2	NA	NA	D		ACTR2		314	C
CHMP1B	NA	NA	D		PLEKHF2	NA	NA	D
GTF2B	NA	NA	D		YWHAZ	315	316, 317	B
PIK3R3		318	C		DBI	NA	NA	D
ATP1B1		319	C		SNRPD2	NA	NA	D
CBX3	236	320, 321	B		RPS27A	NA	NA	D
B2M			A		STAT3			A
PEBP1	322		B		NPM1			A
LSM5	NA	NA	D		SERBP1		329	C
COPS5	323, 324	325	B		GAPDH		330, 331, 332, 333	C
NAP1L1		326, 327, 328	C					

Supplementary table 4: top 10 GO terms HER2time(SC) ranked by p-value.

Analysis performed by using enrichr (https://maayanlab.cloud/Enrichr/) The full outcome of the analysis (including the list of enriched genes) can be found in ***Supplementary/ supplementary table 4 - GO terms HER2time(SC).txt*** at github.com/AndresMCB/DynamicCancerDriver. Alternatively, it can be accessed at https://doi.org/10.6084/m9.figshare.20448384

Term	Overlap	P-value	Adjusted P-value	Old P-value	Old Adjusted P-value	Odds Ratio	Combined Score
positive regulation of transcription, DNA-templated (GO:0045893)	144/1183	2.06E-49	7.43E-46	0	0	5.530837	620.0322
positive regulation of transcription by RNA polymerase II (GO:0045944)	114/908	6.47E-40	1.17E-36	0	0	5.450645	491.8462
regulation of transcription by RNA polymerase II (GO:0006357)	173/2206	1.01E-33	1.21E-30	0	0	3.428122	260.4697
regulation of transcription, DNA-templated (GO:0006355)	172/2244	2.90E-32	2.62E-29	0	0	3.328918	241.7361
negative regulation of transcription, DNA-templated (GO:0045892)	102/948	7.19E-30	5.18E-27	0	0	4.455229	298.9702
regulation of apoptotic process (GO:0042981)	86/742	2.02E-27	1.22E-24	0	0	4.742796	291.5191
positive regulation of nucleic acid-templated transcription (GO:1903508)	70/511	1.06E-26	5.47E-24	0	0	5.634326	336.9725
negative regulation of programmed cell death (GO:0043069)	59/381	2.11E-25	9.50E-23	0	0	6.412707	364.366
negative regulation of cellular macromolecule biosynthetic process (GO:2000113)	70/547	6.56E-25	2.63E-22	0	0	5.199201	289.5087
negative regulation of apoptotic process (GO:0043066)	65/485	2.70E-24	9.74E-22	0	0	5.448538	295.6848

Supplementary table 5: top 10 GO terms VIMtime(SC) ranked by p-value.

Analysis performed by using enrichr (https://maayanlab.cloud/Enrichr/) The full outcome of the analysis (including the list of enriched genes) can be found in ***Supplementary/ supplementary table 5 - GO terms VIMtime(SC).txt*** at github.com/AndresMCB/DynamicCancerDriver. Alternatively, it can be accessed at https://doi.org/10.6084/m9.figshare.20448384

Term	Overlap	P-value	Adjusted P-value	Old P-value	Old Adjusted P-value	Odds Ratio	Combined Score
positive regulation of transcription, DNA-templated (GO:0045893)	125/1183	6.05E-41	1.98E-37	0	0	5.17514	479.2502
regulation of transcription by RNA polymerase II (GO:0006357)	154/2206	2.28E-29	3.73E-26	0	0	3.34034	220.3038
positive regulation of transcription by RNA polymerase II (GO:0045944)	93/908	4.79E-29	4.97E-26	0	0	4.705793	306.8578
negative regulation of transcription, DNA-templated (GO:0045892)	95/948	6.06E-29	4.97E-26	0	0	4.603856	299.1244
regulation of transcription, DNA-templated (GO:0006355)	152/2244	1.60E-27	1.05E-24	0	0	3.210067	198.061
regulation of apoptotic process (GO:0042981)	78/742	4.64E-25	2.54E-22	0	0	4.726716	264.84
negative regulation of transcription by RNA polymerase II (GO:0000122)	73/684	7.26E-24	3.40E-21	0	0	4.769938	254.1402
negative regulation of cellular macromolecule biosynthetic process (GO:2000113)	61/547	5.35E-21	2.19E-18	0	0	4.919179	229.6169
regulation of intracellular signal transduction (GO:1902531)	51/437	1.53E-18	5.57E-16	0	0	5.10016	209.2216
negative regulation of nucleic acid-templated transcription (GO:1903507)	51/464	1.99E-17	6.53E-15	0	0	4.759987	183.047

Supplementary table 6: top 10 GDAs HER2Time(SC) ranked by p-value.

Analysis performed by using enrichr (https://maayanlab.cloud/Enrichr/) The full outcome of the analysis (including the list of enriched genes) can be found in ***Supplementary/ supplementary table 6 - GDAs HER2time(SC).txt*** at github.com/AndresMCB/DynamicCancerDriver. Alternatively, it can be accessed at https://doi.org/10.6084/m9.figshare.20448384

Term	Overlap	P-value	Adjusted P-value	Old P-value	Old Adjusted P-value	Odds Ratio	Combined Score
Mammary Neoplasms	244/2387	1.71E-73	1.12E-69	0	0	5.456696	914.2905
Malignant neoplasm of breast	353/5054	4.62E-69	1.52E-65	0	0	4.396229	691.7339
Breast Carcinoma	348/4963	4.75E-68	1.04E-64	0	0	4.353829	674.9187
Neoplasm Metastasis	292/3920	8.59E-59	1.41E-55	0	0	4.067594	543.8443
Malignant neoplasm of prostate	262/3239	3.30E-58	4.34E-55	0	0	4.225159	559.2211
Tumor Progression	203/2090	2.45E-55	2.68E-52	0	0	4.697222	590.6636
Carcinogenesis	291/4065	1.18E-54	1.11E-51	0	0	3.848428	477.8681
Prostate carcinoma	249/3145	9.09E-53	7.47E-50	0	0	3.996284	478.8745
Malignant neoplasm of pancreas	180/1846	1.85E-48	1.35E-45	0	0	4.517939	496.5704
Non-Small Cell Lung Carcinoma	199/2243	8.76E-48	5.76E-45	0	0	4.171255	451.9703

Supplementary table 7: top 10 GDAs VIMtime(SC) ranked by p-value.

Analysis performed by using enrichr (https://maayanlab.cloud/Enrichr/) The full outcome of the analysis (including the list of enriched genes) can be found in ***Supplementary/ supplementary table 7 - GDAs VIMtime(SC).txt*** at github.com/AndresMCB/DynamicCancerDriver. Alternatively, it can be accessed at https://doi.org/10.6084/m9.figshare.20448384

Term	Overlap	P-value	Adjusted P-value	Old P-value	Old Adjusted P-value	Odds Ratio	Combined Score
Carcinogenesis	273/4065	1.39E-55	8.61E-52	0	0	4.145724	523.6669
Malignant neoplasm of breast	306/5054	1.64E-54	5.08E-51	0	0	3.965856	491.1573
Breast Carcinoma	301/4963	2.31E-53	4.79E-50	0	0	3.914359	474.4149
Malignant neoplasm of prostate	236/3239	3.05E-52	4.74E-49	0	0	4.184243	496.3296
Neoplasm Metastasis	262/3920	3.88E-52	4.81E-49	0	0	3.998027	473.2837
Prostate carcinoma	228/3145	1.27E-49	1.32E-46	0	0	4.077765	459.0921
Malignant neoplasm of ovary	174/2026	8.65E-46	7.67E-43	0	0	4.457805	462.5485
Mammary Neoplasms	190/2387	1.11E-45	8.59E-43	0	0	4.204222	435.1976
Liver carcinoma	236/3593	4.25E-44	2.93E-41	0	0	3.66247	365.7586
Malignant neoplasm of pancreas	162/1846	1.94E-43	1.21E-40	0	0	4.463608	438.9855

Supplementary table 8: Top 100 dynamic cancer drivers from HER2time(Bulk), ranked by frequency of mutation in TCGA (descending order).

Tie break by Relative Causal Impact (descending order). A full comprehensive list with the drivers inferred from HER2time(Bulk) can be found in ***Supplementary/ supplementary table 8 - dynamic cancer drivers HER2time(Bulk).csv*** at github.com/AndresMCB/DynamicCancerDriver. Alternatively, it can be accessed at https://doi.org/10.6084/m9.figshare.20448384

	Ensembl.ID	HGNC.symbol	Mut.Freq	RelEffect	AbsEffect	p
**1**	ENSG00000121879	PIK3CA	328	0.245815	570.8735	0.005
**2**	ENSG00000151914	DST	75	-0.52536	-18758.5	0.005155
**3**	ENSG00000198947	DMD	59	-0.67595	-2818.27	0.015
**4**	ENSG00000039068	CDH1	55	1.973177	20281.88	0.005
**5**	ENSG00000141027	NCOR1	44	0.298908	1743.139	0.005
**6**	ENSG00000127603	MACF1	43	0.961934	10218.31	0.005236
**7**	ENSG00000084674	APOB	42	3.879672	16.24728	0.005
**8**	ENSG00000081479	LRP2	40	5.566193	6432.024	0.005
**9**	ENSG00000085224	ATRX	39	0.520398	2762.932	0.005
**10**	ENSG00000196712	NF1	36	3.157311	6445.952	0.005
**11**	ENSG00000166147	FBN1	34	0.54127	6134.835	0.005181
**12**	ENSG00000171862	PTEN	34	0.451308	2642.034	0.005
**13**	ENSG00000165124	SVEP1	33	-0.81069	-6400.35	0.025
**14**	ENSG00000111642	CHD4	32	-0.09729	-2167.03	0.025
**15**	ENSG00000152818	UTRN	30	0.341521	1549.364	0.015464
**16**	ENSG00000198734	F5	30	-0.53017	-306.427	0.015
**17**	ENSG00000141736	ERBB2	28	19.25144	53328.42	0.005
**18**	ENSG00000065559	MAP2K4	28	0.434703	712.4424	0.005
**19**	ENSG00000196924	FLNA	27	0.434926	25156.14	0.005236
**20**	ENSG00000142208	AKT1	27	-0.66189	-29272.6	0.015
**21**	ENSG00000104517	UBR5	26	0.289933	2360.438	0.015
**22**	ENSG00000149311	ATM	25	0.319154	705.318	0.005236
**23**	ENSG00000178209	PLEC	24	0.635678	10220.11	0.005236
**24**	ENSG00000101680	LAMA1	24	-0.83253	-1963.97	0.01
**25**	ENSG00000178104	PDE4DIP	23	0.695621	2178.394	0.010471
**26**	ENSG00000118058	KMT2A	23	0.438644	1542.594	0.005
**27**	ENSG00000065534	MYLK	23	-0.64402	-13445.6	0.005181
**28**	ENSG00000130396	AFDN	22	1.759308	4982.454	0.015
**29**	ENSG00000278540	ACACA	22	1.11172	3466.663	0.005076
**30**	ENSG00000142798	HSPG2	22	0.527988	6548.406	0.005236
**31**	ENSG00000137076	TLN1	22	0.420664	7233.55	0.015707
**32**	ENSG00000167522	ANKRD11	22	0.120779	506.163	0.025
**33**	ENSG00000196367	TRRAP	22	-0.32451	-3181.78	0.005076
**34**	ENSG00000203710	CR1	22	-0.40821	-65.5736	0.035
**35**	ENSG00000079102	RUNX1T1	22	-0.5674	-488.375	0.025381
**36**	ENSG00000007314	SCN4A	21	2.716321	93.06517	0.005
**37**	ENSG00000169946	ZFPM2	21	2.087188	277.956	0.005236
**38**	ENSG00000159216	RUNX1	21	0.547964	4085.788	0.005
**39**	ENSG00000134250	NOTCH2	21	0.289734	3291.523	0.026178
**40**	ENSG00000079841	RIMS1	20	18.69365	186.2013	0.005
**41**	ENSG00000029534	ANK1	20	3.152989	354.0884	0.005236
**42**	ENSG00000001626	CFTR	20	2.695465	45.47265	0.005
**43**	ENSG00000138162	TACC2	20	2.164844	2471.161	0.005
**44**	ENSG00000110799	VWF	20	1.022028	5815.601	0.005076
**45**	ENSG00000142949	PTPRF	20	0.964981	22105.08	0.005
**46**	ENSG00000197386	HTT	20	-0.35377	-3898.92	0.015464
**47**	ENSG00000225830	ERCC6	20	-0.52358	-1004.93	0.005
**48**	ENSG00000197565	COL4A6	19	679.3059	660.4388	0.005
**49**	ENSG00000107186	MPDZ	19	1.05736	1087.061	0.005
**50**	ENSG00000273079	GRIN2B	19	-0.83304	-115.878	0.035533
**51**	ENSG00000150995	ITPR1	19	-4.6251	6397.099	0.005348
**52**	ENSG00000188153	COL4A5	19	-122.31	3590.755	0.005
**53**	ENSG00000081052	COL4A4	18	0.683564	75.82118	0.005
**54**	ENSG00000012048	BRCA1	18	0.469256	395.4063	0.015
**55**	ENSG00000064012	CASP8	18	0.451831	502.2222	0.036082
**56**	ENSG00000131626	PPFIA1	18	0.434158	1626.502	0.005076
**57**	ENSG00000107485	GATA3	18	-27.3602	38154.09	0.005
**58**	ENSG00000174469	CNTNAP2	17	65.65806	1993.775	0.005
**59**	ENSG00000147255	IGSF1	17	29.03406	1806.542	0.005236
**60**	ENSG00000185008	ROBO2	17	3.575056	554.7719	0.015707
**61**	ENSG00000146648	EGFR	17	1.729244	3156.434	0.005
**62**	ENSG00000018236	CNTN1	17	1.566935	485.53	0.015
**63**	ENSG00000112282	MED23	17	1.517827	2351.494	0.005
**64**	ENSG00000170776	AKAP13	17	0.900338	4075.433	0.005
**65**	ENSG00000197081	IGF2R	17	0.300825	2326.957	0.005236
**66**	ENSG00000100393	EP300	17	0.2657	1560.264	0.045
**67**	ENSG00000102974	CTCF	17	0.189471	715.382	0.01
**68**	ENSG00000132694	ARHGEF11	17	-0.23254	-1560.98	0.015
**69**	ENSG00000153201	RANBP2	17	-0.35645	-6457.65	0.025
**70**	ENSG00000178568	ERBB4	16	273.4745	2705.585	0.005
**71**	ENSG00000089250	NOS1	16	2.853439	14.08916	0.005
**72**	ENSG00000198753	PLXNB3	16	2.332504	1045.554	0.005236
**73**	ENSG00000141367	CLTC	16	0.809977	19211.59	0.005
**74**	ENSG00000167306	MYO5B	16	0.790746	2490.094	0.005
**75**	ENSG00000197694	SPTAN1	16	0.513481	8191.207	0.005236
**76**	ENSG00000114127	XRN1	16	0.427979	809.9177	0.005
**77**	ENSG00000049759	NEDD4L	16	0.361481	1342.941	0.005
**78**	ENSG00000111859	NEDD9	16	-0.34058	-1786.02	0.025
**79**	ENSG00000196313	POM121	16	-0.40287	-1549.11	0.04
**80**	ENSG00000169855	ROBO1	16	-0.45522	-2649.16	0.015
**81**	ENSG00000077522	ACTN2	16	-0.54663	-159.909	0.04
**82**	ENSG00000182901	RGS7	15	109.5107	41.92506	0.005236
**83**	ENSG00000183454	GRIN2A	15	86.18357	125.366	0.015707
**84**	ENSG00000078328	RBFOX1	15	12.53668	5.686938	0.005208
**85**	ENSG00000155511	GRIA1	15	6.868589	253.2287	0.005236
**86**	ENSG00000174175	SELP	15	1.223755	283.5931	0.005
**87**	ENSG00000112818	MEP1A	15	0.986936	3.173108	0.01
**88**	ENSG00000132849	PATJ	15	0.95111	3079.854	0.015
**89**	ENSG00000151276	MAGI1	15	0.408507	675.4461	0.005
**90**	ENSG00000130985	UBA1	15	-0.23857	-8362.73	0.005
**91**	ENSG00000196083	IL1RAP	15	-0.48773	-762.174	0.005155
**92**	ENSG00000197535	MYO5A	15	-0.68703	-5707.46	0.005181
**93**	ENSG00000133124	IRS4	14	412.3778	117.6291	0.005025
**94**	ENSG00000148053	NTRK2	14	3.167965	3245.432	0.005236
**95**	ENSG00000065618	COL17A1	14	2.123479	1853.503	0.005076
**96**	ENSG00000182752	PAPPA	14	2.048972	161.1572	0.005236
**97**	ENSG00000106571	GLI3	14	0.827262	1776.633	0.005
**98**	ENSG00000100852	ARHGAP5	14	0.727277	2731.178	0.005236
**99**	ENSG00000124151	NCOA3	14	0.684853	1772.362	0.005
**100**	ENSG00000112851	ERBIN	14	0.527422	3142.484	0.005181

Supplementary table 9: Top 100 dynamic cancer drivers from VIMtime(Bulk), ranked by frequency of mutation in TCGA (descending order).

Tie break by Relative Causal Impact (descending order). A full comprehensive list with the drivers inferred from VIMtime(Bulk) can be found in ***supplementary table 9 - dynamic cancer drivers VIMtime(Bulk).csv*** at github.com/AndresMCB/DynamicCancerDriver. Alternatively, it can be accessed at https://doi.org/10.6084/m9.figshare.20448384

	Ensembl.ID	HGNC.symbol	Mut.Freq	RelEffect	AbsEffect	p			Ensembl.ID	HGNC.symbol	Mut.Freq	RelEffect	AbsEffect	p
**1**	ENSG00000121879	PIK3CA	328	0.345788	744.0998	0.005		51	ENSG00000139687	RB1	20	0.49751	1475.884	0.005076
**2**	ENSG00000198626	RYR2	85	4.207954	121.5235	0.005		52	ENSG00000138162	TACC2	20	0.41903	1064.017	0.005
**3**	ENSG00000151914	DST	75	0.810778	7603.924	0.005		53	ENSG00000112769	LAMA4	20	0.41313	1759.742	0.03
**4**	ENSG00000163554	SPTA1	57	0.843172	12.33052	0.005025		54	ENSG00000198646	NCOA6	20	0.28724	1181.123	0.005236
**5**	ENSG00000039068	CDH1	55	1.04397	15585.82	0.005		55	ENSG00000142949	PTPRF	20	-0.43415	-34519.6	0.025773
**6**	ENSG00000127603	MACF1	43	1.600195	12841.21	0.005		56	ENSG00000176406	RIMS2	19	67.2837	240.5586	0.005
**7**	ENSG00000084674	APOB	42	37.69888	19.92137	0.005		57	ENSG00000197565	COL4A6	19	9.16545	594.5745	0.03
**8**	ENSG00000081479	LRP2	40	2.038013	5082.172	0.005		58	ENSG00000273079	GRIN2B	19	3.78143	18.91918	0.02
**9**	ENSG00000085224	ATRX	39	0.359272	2133.592	0.005236		59	ENSG00000107186	MPDZ	19	0.93998	1025.01	0.005
**10**	ENSG00000127914	AKAP9	37	-0.66225	-21957.5	0.005		60	ENSG00000188153	COL4A5	19	0.58	1303.352	0.015
**11**	ENSG00000196712	NF1	36	5.059854	7075.278	0.005236		61	ENSG00000137474	MYO7A	19	0.31211	134.4706	0.03
**12**	ENSG00000065526	SPEN	35	1.822539	4250.23	0.005		62	ENSG00000082898	XPO1	19	0.14889	1508.017	0.005236
**13**	ENSG00000166147	FBN1	34	7.633048	15482.57	0.005		63	ENSG00000128591	FLNC	19	-0.48673	-1134.23	0.020725
**14**	ENSG00000165124	SVEP1	33	1.062377	773.841	0.005		64	ENSG00000142449	FBN3	19	-1.54672	589.6525	0.02
**15**	ENSG00000111642	CHD4	32	0.205198	3429.26	0.025		65	ENSG00000161681	SHANK1	19	-3.55523	80.60168	0.020942
**16**	ENSG00000152818	UTRN	30	9.464905	5506.286	0.005		66	ENSG00000081052	COL4A4	18	30.1425	181.2441	0.005
**17**	ENSG00000198734	F5	30	2.377713	193.7885	0.02		67	ENSG00000067369	TP53BP1	18	1.91657	2220.119	0.005
**18**	ENSG00000153707	PTPRD	30	-2.2059	1508.761	0.04		68	ENSG00000131626	PPFIA1	18	0.94291	2609.534	0.005
**19**	ENSG00000123384	LRP1	29	9.982512	24709.41	0.005		69	ENSG00000012048	BRCA1	18	0.59592	461.7295	0.045685
**20**	ENSG00000166963	MAP1A	29	3.573477	1302.94	0.005		70	ENSG00000094631	HDAC6	18	0.47688	1059.178	0.005
**21**	ENSG00000065559	MAP2K4	28	0.746543	1001.479	0.04		71	ENSG00000147162	OGT	18	-0.39422	-6080.94	0.020942
**22**	ENSG00000142208	AKT1	27	0.514225	5081.257	0.005236		72	ENSG00000146648	EGFR	17	6.012	4279.453	0.005236
**23**	ENSG00000185010	F8	27	-0.59579	-1480.95	0.02		73	ENSG00000147255	IGSF1	17	4.36987	1519.285	0.005236
**24**	ENSG00000042832	TG	26	4.192094	70.0546	0.005		74	ENSG00000163629	PTPN13	17	2.86052	2126.187	0.005
**25**	ENSG00000149311	ATM	25	4.562618	2396.868	0.005		75	ENSG00000106278	PTPRZ1	17	2.689	210.4911	0.020942
**26**	ENSG00000101680	LAMA1	24	4.36995	330.5575	0.005		76	ENSG00000018236	CNTN1	17	2.47952	566.872	0.045
**27**	ENSG00000127329	PTPRB	24	1.833719	1538.255	0.005		77	ENSG00000169031	COL4A3	17	2.3773	50.87343	0.005
**28**	ENSG00000178209	PLEC	24	0.554149	9368.297	0.005076		78	ENSG00000174469	CNTNAP2	17	1.27211	1129.258	0.005
**29**	ENSG00000038427	VCAN	23	2.048457	16525.61	0.005		79	ENSG00000100393	EP300	17	1.22172	4093.092	0.005155
**30**	ENSG00000134982	APC	23	1.559689	1346.885	0.020942		80	ENSG00000185008	ROBO2	17	0.65907	281.5608	0.040404
**31**	ENSG00000065534	MYLK	23	1.243209	4132.391	0.005208		81	ENSG00000102974	CTCF	17	0.62882	1733.975	0.005
**32**	ENSG00000118058	KMT2A	23	0.889178	2384.59	0.005		82	ENSG00000112282	MED23	17	0.39046	1095.503	0.005
**33**	ENSG00000142798	HSPG2	22	14.47531	17764.88	0.005		83	ENSG00000153201	RANBP2	17	0.29958	2693.065	0.005
**34**	ENSG00000100503	NIN	22	1.540668	2161.873	0.005		84	ENSG00000101868	POLA1	17	0.20408	263.3489	0.020619
**35**	ENSG00000130396	AFDN	22	0.995157	3900.523	0.005236		85	ENSG00000197081	IGF2R	17	-0.57152	-13446.8	0.005155
**36**	ENSG00000096433	ITPR3	22	0.843371	2741.744	0.005155		86	ENSG00000170776	AKAP13	17	-76.2496	8725.715	0.020942
**37**	ENSG00000196367	TRRAP	22	0.759431	2868.675	0.005		87	ENSG00000198932	GPRASP1	16	211.617	435.8121	0.005236
**38**	ENSG00000167522	ANKRD11	22	0.504255	1578.139	0.005155		88	ENSG00000077522	ACTN2	16	3.00835	100.4633	0.005
**39**	ENSG00000137076	TLN1	22	0.406877	7075.517	0.02		89	ENSG00000164692	COL1A2	16	1.73385	174362.1	0.005
**40**	ENSG00000079102	RUNX1T1	22	-0.84228	-1998.55	0.015152		90	ENSG00000089250	NOS1	16	1.45626	11.26545	0.005
**41**	ENSG00000169946	ZFPM2	21	17.5438	389.4492	0.005		91	ENSG00000198753	PLXNB3	16	1.02051	752.7958	0.010309
**42**	ENSG00000159216	RUNX1	21	12.13214	10676.8	0.02		92	ENSG00000136869	TLR4	16	0.93304	475.4087	0.005
**43**	ENSG00000134250	NOTCH2	21	2.218413	10127.18	0.005236		93	ENSG00000145675	PIK3R1	16	0.71824	3417.801	0.005
**44**	ENSG00000007314	SCN4A	21	1.620168	78.69703	0.005		94	ENSG00000141367	CLTC	16	0.71262	17864.83	0.005
**45**	ENSG00000029534	ANK1	20	32.53428	452.8636	0.005236		95	ENSG00000197694	SPTAN1	16	0.58407	8909.812	0.015
**46**	ENSG00000001626	CFTR	20	10.93548	57.16355	0.005		96	ENSG00000114127	XRN1	16	0.51134	914.64	0.005
**47**	ENSG00000225830	ERCC6	20	4.851821	756.8204	0.031414		97	ENSG00000167306	MYO5B	16	0.37732	1542.141	0.020942
**48**	ENSG00000079841	RIMS1	20	1.219106	107.5541	0.005		98	ENSG00000139618	BRCA2	16	0.29356	212.2813	0.03
**49**	ENSG00000110799	VWF	20	0.758277	4968.041	0.005076		99	ENSG00000085511	MAP3K4	16	0.22794	358.4106	0.031414
**50**	ENSG00000070961	ATP2B1	20	0.508201	1021.101	0.005		100	ENSG00000263528	IKBKE	16	-0.21876	-329.815	0.04712

Supplementary table 10: DCD(HER2_SC) Reg. relationships. Regulatory relationships between the top 200 of DCDs and the set (not restricted to the top 200 DCDs) of CGC Breast Cancer drivers detected by our method (when using "HER2time(SC)"). BRCA-TCGA Regulatory relationships extracted from http://www.grndb.com/

	TF	TF.isCGC	gene	gene.isCGC	NES	Confidence		TF	TF.isCGC	gene	gene.isCGC	NES	Confidence
**1**	BCL3	FALSE	BCL3	FALSE	5.07	High	**42**	E2F2	FALSE	CDK1	FALSE	11	High
**2**	BCL3	FALSE	GNB2	FALSE	4.1	High	**43**	E2F2	FALSE	FEN1	TRUE	11	High
**3**	BCL3	FALSE	PRKACA	FALSE	4.1	High	**44**	E2F2	FALSE	MCM6	FALSE	11	High
**4**	BCL3	FALSE	RGS16	FALSE	4.1	High	**45**	E2F2	FALSE	MYBL2	FALSE	11	High
**5**	BRCA1	TRUE	MED1	FALSE	5.29	High	**46**	E2F2	FALSE	SF3B3	FALSE	11	High
**6**	BRCA1	TRUE	RFC5	FALSE	5.29	High	**47**	E2F2	FALSE	BRCA2	TRUE	10.9	High
**7**	BRCA1	TRUE	BRCA1	TRUE	5.22	High	**48**	E2F2	FALSE	CHEK2	TRUE	10.9	High
**8**	BRCA1	TRUE	MELK	FALSE	5.22	High	**49**	E2F2	FALSE	HTATSF1	FALSE	10.9	High
**9**	BRCA1	TRUE	TTF2	FALSE	5.22	High	**50**	E2F2	FALSE	MELK	FALSE	10.9	High
**10**	BRCA1	TRUE	E2F2	FALSE	5.19	High	**51**	E2F2	FALSE	BRCA1	TRUE	10.7	High
**11**	BRCA1	TRUE	CCND1	TRUE	4.82	High	**52**	E2F2	FALSE	EXOSC2	FALSE	10.7	High
**12**	BRCA1	TRUE	CDC25B	FALSE	4.82	High	**53**	E2F2	FALSE	MTA2	FALSE	10.7	High
**13**	BRCA1	TRUE	HTT	FALSE	4.82	High	**54**	E2F2	FALSE	RFC5	FALSE	10.7	High
**14**	BRCA1	TRUE	MYBL2	FALSE	4.82	High	**55**	E2F2	FALSE	TTF2	FALSE	10.7	High
**15**	BRCA1	TRUE	STAT5B	FALSE	4.14	High	**56**	ESR1	TRUE	ERBB4	FALSE	4.28	High
**16**	BRCA1	TRUE	BRCA2	TRUE	4.1	High	**57**	ESR1	TRUE	NRIP1	FALSE	4.28	High
**17**	BRCA1	TRUE	MCM6	FALSE	4.1	High	**58**	ESR1	TRUE	ESR1	TRUE	4.15	High
**18**	BRCA1	TRUE	TRIM37	FALSE	4.1	High	**59**	ESR1	TRUE	GAK	FALSE	3.8	High
**19**	BRCA1	TRUE	CENPJ	FALSE	3.93	High	**60**	ESR1	TRUE	IKBKB	FALSE	3.53	High
**20**	BRCA1	TRUE	CHEK2	TRUE	3.92	High	**61**	ESR1	TRUE	ERBB2	TRUE	3.1	High
**21**	BRCA1	TRUE	EXOSC2	FALSE	3.92	High	**62**	HOXB7	FALSE	HOXB7	FALSE	4.23	High
**22**	BRCA1	TRUE	PIP4K2C	FALSE	3.66	High	**63**	MYBL2	FALSE	CENPJ	FALSE	3.05	High
**23**	BRCA1	TRUE	PLK1	FALSE	3.37	High	**64**	MYBL2	FALSE	EXOSC2	FALSE	3.05	High
**24**	CTCF	TRUE	CDH1	TRUE	3.26	High	**65**	MYBL2	FALSE	MTA2	FALSE	3.05	High
**25**	CTCF	TRUE	CSNK2A2	FALSE	3.26	High	**66**	MYBL2	FALSE	RFC5	FALSE	3.05	High
**26**	CTCF	TRUE	CTCF	TRUE	3.26	High	**67**	NFATC2	FALSE	CYFIP2	FALSE	3.71	High
**27**	E2F2	FALSE	CDC25B	FALSE	9.88	High	**68**	NFATC2	FALSE	IL16	FALSE	3.71	High
**28**	E2F2	FALSE	CENPJ	FALSE	9.88	High	**69**	NFATC2	FALSE	ITK	FALSE	3.71	High
**29**	E2F2	FALSE	MDC1	FALSE	8.87	High	**70**	RB1	TRUE	RB1	TRUE	3.24	High
**30**	E2F2	FALSE	PLK1	FALSE	8.59	High	**71**	SOX9	FALSE	REPS1	FALSE	3.8	High
**31**	E2F2	FALSE	CCT7	FALSE	8.58	High	**72**	SOX9	FALSE	SOX9	FALSE	3.8	High
**32**	E2F2	FALSE	CDKN2A	FALSE	8.58	High	**73**	STAT5B	FALSE	STAT5B	FALSE	3.02	High
**33**	E2F2	FALSE	MPHOSPH6	FALSE	8.58	High	**74**	THRA	FALSE	ERBB2	TRUE	5.67	High
**34**	E2F2	FALSE	TAF4	FALSE	8.58	High	**75**	THRA	FALSE	GRB7	FALSE	5.67	High
**35**	E2F2	FALSE	TRIM37	FALSE	8.58	High	**76**	THRA	FALSE	THRA	FALSE	5.67	High
**36**	E2F2	FALSE	PTPN2	FALSE	8.28	High	**77**	THRA	FALSE	STAT5B	FALSE	4.97	High
**37**	E2F2	FALSE	BAG6	FALSE	7.8	High	**78**	THRA	FALSE	NFE2L1	FALSE	3.79	High
**38**	E2F2	FALSE	PLCG1	FALSE	7.34	High	**79**	THRA	FALSE	PLXNB1	FALSE	3.79	High
**39**	E2F2	FALSE	PIK3R2	FALSE	6.86	High	**80**	THRA	FALSE	UNC119	FALSE	3.79	High
**40**	E2F2	FALSE	LSM2	FALSE	5.82	High	**81**	THRA	FALSE	PKN1	FALSE	3.09	High
**41**	E2F2	FALSE	E2F2	FALSE	11.2	High							

Supplementary table 11: DCD(HER2_Bulk) Reg. relationships. Regulatory relationships between the top 200 of DCDs and the set (not restricted to the top 200 DCDs) of CGC Breast Cancer drivers detected by our method (when using “HER2time(Bulk)”). BRCA-TCGA Regulatory relationships extracted from http://www.grndb.com/

	TF	TF.isCGC	gene	gene.isCGC	NES	Confidence		TF	TF.isCGC	gene	gene.isCGC	NES	Confidence
**1**	BRCA1	TRUE	BRCA1	TRUE	5.22	High	**29**	KMT2A	FALSE	EP300	TRUE	3.03	High
**2**	BRCA1	TRUE	CCND1	TRUE	4.82	High	**30**	KMT2A	FALSE	SMARCA2	FALSE	3.03	High
**3**	BRCA1	TRUE	CELSR3	FALSE	4.82	High	**31**	KMT2A	FALSE	XRN1	FALSE	3.03	High
**4**	BRCA1	TRUE	HTT	FALSE	4.82	High	**32**	RFX3	FALSE	FGFR2	FALSE	7.33	High
**5**	BRCA1	TRUE	SMARCC1	FALSE	4.56	High	**33**	RFX3	FALSE	MPDZ	FALSE	7.33	High
**6**	BRCA1	TRUE	BARD1	TRUE	3.93	High	**34**	RFX3	FALSE	RFX3	FALSE	7.33	High
**7**	BRCA1	TRUE	PARP1	FALSE	3.92	High	**35**	RFX3	FALSE	SMARCA2	FALSE	7.33	High
**8**	BRCA1	TRUE	RFC1	FALSE	3.92	High	**36**	RFX3	FALSE	MAGI1	FALSE	7.32	High
**9**	CTCF	TRUE	CDH1	TRUE	3.26	High	**37**	RFX3	FALSE	CDKN1B	TRUE	6.77	High
**10**	CTCF	TRUE	CTCF	TRUE	3.26	High	**38**	RFX3	FALSE	ATP2B4	FALSE	6.17	High
**11**	EP300	TRUE	EP300	TRUE	3.42	High	**39**	RFX3	FALSE	NTRK2	FALSE	6.17	High
**12**	ESR1	TRUE	ITPR1	FALSE	4.37	High	**40**	RFX3	FALSE	SORBS2	FALSE	6.17	High
**13**	ESR1	TRUE	WWP1	FALSE	4.37	High	**41**	RFX3	FALSE	COL4A5	FALSE	5.38	High
**14**	ESR1	TRUE	ERBB4	FALSE	4.28	High	**42**	RFX3	FALSE	GLI3	FALSE	3.08	High
**15**	ESR1	TRUE	NEDD4L	FALSE	4.28	High	**43**	RUNX1	FALSE	RUNX1	FALSE	4.08	High
**16**	ESR1	TRUE	ESR1	TRUE	4.15	High	**44**	SIN3A	FALSE	SIN3A	FALSE	4.23	High
**17**	ESR1	TRUE	SPTBN4	FALSE	3.86	High	**45**	SIN3A	FALSE	DOCK1	FALSE	3.7	High
**18**	ESR1	TRUE	IKBKB	FALSE	3.53	High	**46**	SIN3A	FALSE	IGF1R	FALSE	3.7	High
**19**	ESR1	TRUE	GATA3	TRUE	3.36	High	**47**	SIN3A	FALSE	CHD4	FALSE	3.03	High
**20**	ESR1	TRUE	ERBB2	TRUE	3.1	High	**48**	SIN3A	FALSE	MAGI3	FALSE	3.03	High
**21**	ESR1	TRUE	ITGA3	FALSE	3.1	High	**49**	SIN3A	FALSE	PER2	FALSE	3.03	High
**22**	ESR1	TRUE	FGFR2	FALSE	3.08	High	**50**	SIN3A	FALSE	RFC1	FALSE	3.03	High
**23**	ESRRG	FALSE	ESRRG	FALSE	4.18	High	**51**	STAT5A	FALSE	STAT5A	FALSE	4.25	High
**24**	ESRRG	FALSE	SORBS2	FALSE	4.15	High	**52**	TP63	FALSE	COL17A1	FALSE	4.94	High
**25**	ESRRG	FALSE	CNTN2	FALSE	3.23	High	**53**	TP63	FALSE	DST	FALSE	3.69	High
**26**	GATA3	TRUE	GATA3	TRUE	5.62	High	**54**	TP63	FALSE	TP63	FALSE	3.69	High
**27**	GATA3	TRUE	ESR1	TRUE	3.14	High	**55**	TP63	FALSE	COL4A6	FALSE	3.22	High
**28**	KMT2A	FALSE	ATM	FALSE	3.34	High	**56**	TP63	FALSE	GRIA4	FALSE	3	High

Supplementary table 12: Top 100 dynamic cancer drivers from ESR1time(SC), ranked by Relative Causal Impact (descending order).

A full comprehensive list with the drivers inferred from HER2time(Bulk) can be found in ***Supplementary/ supplementary table 12 - dynamic cancer drivers ESR1time(SC).csv*** at github.com/AndresMCB/DynamicCancerDriver. Alternatively, it can be accessed at https://doi.org/10.6084/m9.figshare.20448384

	Ensembl.ID	HGNC.symbol	RelEffect	AbsEffect	p
**1**	ENSG00000091831	ESR1	1653.320713	54.1638264	0.005464481
**2**	ENSG00000037965	HOXC8	76.12010664	1.200778119	0.005464481
**3**	ENSG00000182866	LCK	55.00766248	4.139993843	0.005464481
**4**	ENSG00000175899	A2M	53.65517696	137.214403	0.005464481
**5**	ENSG00000081479	LRP2	40.43558545	9.418973532	0.005464481
**6**	ENSG00000119335	SET	37.01455589	655.5826807	0.021857923
**7**	ENSG00000113140	SPARC	36.3955162	13.86551692	0.005464481
**8**	ENSG00000187098	MITF	19.07612718	13.77239825	0.005464481
**9**	ENSG00000169439	SDC2	19.0487882	23.53049286	0.005464481
**10**	ENSG00000157110	RBPMS	15.44111011	18.27926647	0.021857923
**11**	ENSG00000109846	CRYAB	14.10751587	102.9105574	0.005464481
**12**	ENSG00000149091	DGKZ	13.89991909	3.470302376	0.005464481
**13**	ENSG00000119535	CSF3R	13.19261251	13.50083471	0.005464481
**14**	ENSG00000126351	THRA	9.242090372	6.958917839	0.005464481
**15**	ENSG00000108599	AKAP10	8.305777415	13.63136856	0.007692308
**16**	ENSG00000136936	XPA	7.216224659	5.212813635	0.005
**17**	ENSG00000131759	RARA	6.832735415	4.779657592	0.005464481
**18**	ENSG00000079819	EPB41L2	6.640824534	12.73753238	0.005464481
**19**	ENSG00000067715	SYT1	6.197226864	10.16873756	0.005464481
**20**	ENSG00000157404	KIT	5.524654499	9.207739349	0.005
**21**	ENSG00000065534	MYLK	5.503180266	17.32245371	0.005464481
**22**	ENSG00000168078	PBK	5.489619456	8.911739297	0.005464481
**23**	ENSG00000140443	IGF1R	5.260535291	30.31809033	0.005464481
**24**	ENSG00000131747	TOP2A	5.256607597	175.6460702	0.005464481
**25**	ENSG00000109339	MAPK10	5.179064068	16.45709114	0.005464481
**26**	ENSG00000165140	FBP1	4.93810487	34.69870218	0.005464481
**27**	ENSG00000113739	STC2	4.577167106	9.205284894	0.005464481
**28**	ENSG00000118785	SPP1	4.53609166	53.3005324	0.032786885
**29**	ENSG00000105048	TNNT1	4.263696276	25.72583017	0.005464481
**30**	ENSG00000087586	AURKA	4.105410442	11.41849972	0.007692308
**31**	ENSG00000127445	PIN1	3.229827462	4.92938909	0.005464481
**32**	ENSG00000129691	ASH2L	3.179787327	15.38251119	0.005464481
**33**	ENSG00000077782	FGFR1	3.131685267	31.36779476	0.005464481
**34**	ENSG00000107281	NPDC1	3.105415137	6.817273885	0.005464481
**35**	ENSG00000145604	SKP2	3.049140361	7.221493253	0.005464481
**36**	ENSG00000170004	CHD3	3.02556319	3.860081851	0.005464481
**37**	ENSG00000136997	MYC	2.889315153	59.36452415	0.005464481
**38**	ENSG00000151208	DLG5	2.741503129	17.96605903	0.005464481
**39**	ENSG00000123143	PKN1	2.73248975	2.540375617	0.005464481
**40**	ENSG00000071539	TRIP13	2.562377621	3.240800215	0.049180328
**41**	ENSG00000097007	ABL1	2.479609878	3.13916293	0.005464481
**42**	ENSG00000120696	KBTBD7	2.41600395	3.7339964	0.005464481
**43**	ENSG00000110092	CCND1	2.359680704	27.33129896	0.049180328
**44**	ENSG00000177082	WDR73	2.337024585	11.88491824	0.007042254
**45**	ENSG00000079462	PAFAH1B3	2.30924976	43.82234123	0.005464481
**46**	ENSG00000103168	TAF1C	2.235673	1.619028649	0.005
**47**	ENSG00000120837	NFYB	2.178287274	15.86348939	0.005464481
**48**	ENSG00000134057	CCNB1	2.064575117	32.15039567	0.005464481
**49**	ENSG00000196470	SIAH1	2.029859483	18.09953377	0.005464481
**50**	ENSG00000101017	CD40	1.957145542	3.719744517	0.005464481

	Ensembl.ID	HGNC.symbol	RelEffect	AbsEffect	p
**51**	ENSG00000171791	BCL2	1.93272065	3.855197704	0.043715847
**52**	ENSG00000109472	CPE	1.82054402	29.77971336	0.005464481
**53**	ENSG00000109103	UNC119	1.78142162	11.54536444	0.005464481
**54**	ENSG00000197943	PLCG2	1.72712222	1.709664369	0.005464481
**55**	ENSG00000184634	MED12	1.72059475	3.158475573	0.005464481
**56**	ENSG00000133740	E2F5	1.72042433	6.143056534	0.005464481
**57**	ENSG00000164733	CTSB	1.69875058	94.36181526	0.005464481
**58**	ENSG00000070770	CSNK2A2	1.62604837	23.18222399	0.005464481
**59**	ENSG00000077348	EXOSC5	1.60972092	17.74000138	0.005464481
**60**	ENSG00000204371	EHMT2	1.60842368	13.67562266	0.005464481
**61**	ENSG00000170310	STX8	1.60461927	10.58394227	0.005464481
**62**	ENSG00000127511	SIN3B	1.60136916	2.040009255	0.005464481
**63**	ENSG00000141456	PELP1	1.55643484	1.442241959	0.005464481
**64**	ENSG00000100083	GGA1	1.55032126	7.285332704	0.049180328
**65**	ENSG00000113716	HMGXB3	1.41801845	1.943732485	0.005464481
**66**	ENSG00000113522	RAD50	1.38382315	17.02094497	0.005464481
**67**	ENSG00000163629	PTPN13	1.36427953	5.718723975	0.027322404
**68**	ENSG00000167110	GOLGA2	1.33848197	9.984944366	0.005
**69**	ENSG00000077942	FBLN1	1.32240165	7.334386279	0.021857923
**70**	ENSG00000196150	ZNF250	1.31508907	7.786895946	0.021857923
**71**	ENSG00000111674	ENO2	1.29308574	8.51681511	0.016393443
**72**	ENSG00000121022	COPS5	1.28089853	54.11795698	0.005235602
**73**	ENSG00000132382	MYBBP1A	1.27735534	5.019447694	0.005464481
**74**	ENSG00000135698	MPHOSPH6	1.24955468	38.44519998	0.010928962
**75**	ENSG00000213281	NRAS	1.24936558	4.553390261	0.023076923
**76**	ENSG00000087266	SH3BP2	1.23422872	1.611251322	0.005464481
**77**	ENSG00000185122	HSF1	1.22103992	3.314173687	0.005464481
**78**	ENSG00000136068	FLNB	1.16460434	21.90237202	0.005464481
**79**	ENSG00000135047	CTSL	1.15807297	24.13652542	0.005464481
**80**	ENSG00000124357	NAGK	1.1329771	40.70216559	0.005464481
**81**	ENSG00000126247	CAPNS1	1.11812916	114.3972215	0.005464481
**82**	ENSG00000103479	RBL2	1.1135723	13.86892962	0.027322404
**83**	ENSG00000108821	COL1A1	1.10332829	1.600024705	0.038251366
**84**	ENSG00000178209	PLEC	1.09872466	1.360920126	0.025
**85**	ENSG00000115461	IGFBP5	1.07997006	8.362817778	0.005464481
**86**	ENSG00000126934	MAP2K2	1.0739435	3.061540419	0.038251366
**87**	ENSG00000104365	IKBKB	1.04883459	9.101406615	0.021857923
**88**	ENSG00000071894	CPSF1	1.00718689	22.35645291	0.027322404
**89**	ENSG00000071051	NCK2	1.00706383	3.373263964	0.027322404
**90**	ENSG00000260027	HOXB7	0.99534307	4.370169094	0.016393443
**91**	ENSG00000124171	PARD6B	0.95655364	85.51252931	0.005464481
**92**	ENSG00000163684	RPP14	0.93286011	12.97206056	0.005
**93**	ENSG00000134352	IL6ST	0.91539684	35.36825286	0.005464481
**94**	ENSG00000254505	CHMP4A	0.91438683	23.94276557	0.005464481
**95**	ENSG00000177700	POLR2L	0.87959436	42.16409312	0.010928962
**96**	ENSG00000175324	LSM1	0.86655725	38.35851341	0.005464481
**97**	ENSG00000015171	ZMYND11	0.8561764	13.03135993	0.005464481
**98**	ENSG00000135046	ANXA1	0.84093856	81.02605443	0.01
**99**	ENSG00000197442	MAP3K5	0.83851295	2.960315646	0.021857923
**100**	ENSG00000078061	ARAF	0.82709002	7.420131098	0.021857923

Supplementary table 13: Top 100 dynamic cancer drivers from ESR1time(Bulk), ranked by frequency of mutation in TCGA (descending order).

Tie break by Relative Causal Impact (descending order). A full comprehensive list with the drivers inferred from ESR1time(Bulk) can be found in ***Supplementary/ supplementary table 13 - dynamic cancer drivers ESR1time(Bulk).csv*** at github.com/AndresMCB/DynamicCancerDriver. Alternatively, it can be accessed at https://doi.org/10.6084/m9.figshare.20448384

	Ensembl.ID	HGNC.symbol	mut. Freq.	RelEffect	AbsEffect	p		Ensembl.ID	HGNC.symbol	mut. Freq.	RelEffect	AbsEffect	p
**1**	ENSG00000155657	TTN	335	0.4313709	320.7028247	0.005882353	**51**	ENSG00000155511	GRIA1	15	48.30982	344.4881	0.005
**2**	ENSG00000141027	NCOR1	44	0.2017683	1338.348049	0.015384615	**52**	ENSG00000078328	RBFOX1	15	3.96935	5.600472	0.005
**3**	ENSG00000084674	APOB	42	1.3337253	13.09518103	0.005	**53**	ENSG00000137497	NUMA1	15	0.641327	9473.655	0.015385
**4**	ENSG00000081479	LRP2	40	3.336767	6833.985672	0.005	**54**	ENSG00000174175	SELP	15	0.545722	197.0031	0.010471
**5**	ENSG00000085224	ATRX	39	0.1603189	1161.862157	0.01	**55**	ENSG00000132849	PATJ	15	0.329826	1687.53	0.005
**6**	ENSG00000127914	AKAP9	37	0.3989218	3476.966395	0.015384615	**56**	ENSG00000151276	MAGI1	15	0.2002	408.7268	0.015
**7**	ENSG00000143669	LYST	36	0.2607073	854.3103788	0.030769231	**57**	ENSG00000197535	MYO5A	15	-0.21375	-709.586	0.030769
**8**	ENSG00000065526	SPEN	35	0.1873329	1066.11487	0.01	**58**	ENSG00000049323	LTBP1	15	-0.27125	-1670.49	0.026178
**9**	ENSG00000171862	PTEN	34	0.1855387	1383.038363	0.04	**59**	ENSG00000148053	NTRK2	14	1.600647	2914.674	0.005
**10**	ENSG00000152818	UTRN	30	0.2589171	1339.465307	0.005235602	**60**	ENSG00000106571	GLI3	14	1.311216	2543.186	0.005236
**11**	ENSG00000166963	MAP1A	29	0.4006653	516.5232853	0.03	**61**	ENSG00000183337	BCOR	14	0.426438	1211.062	0.005
**12**	ENSG00000065559	MAP2K4	28	0.3225901	604.5934903	0.015384615	**62**	ENSG00000107554	DNMBP	14	0.37005	567.1205	0.005
**13**	ENSG00000185010	F8	27	0.2841896	231.5473959	0.02	**63**	ENSG00000158301	GPRASP2	14	0.267256	140.0142	0.04
**14**	ENSG00000157106	SMG1	27	-0.1021287	-1041.839094	0.041884817	**64**	ENSG00000160294	MCM3AP	14	0.213435	1113.668	0.005236
**15**	ENSG00000142208	AKT1	27	-0.3218988	-7258.736435	0.005882353	**65**	ENSG00000112851	ERBIN	14	0.151901	1277.773	0.030769
**16**	ENSG00000104517	UBR5	26	0.205938	1820.078304	0.04	**66**	ENSG00000102081	FMR1	14	-0.14281	-751.002	0.035
**17**	ENSG00000115414	FN1	22	-0.2403115	-80430.1553	0.046153846	**67**	ENSG00000124151	NCOA3	14	-0.20952	-1177.49	0.009091
**18**	ENSG00000065361	ERBB3	21	0.5372075	8970.038519	0.005	**68**	ENSG00000160460	SPTBN4	13	3.767501	398.8827	0.005
**19**	ENSG00000159216	RUNX1	21	0.2869351	2729.850432	0.005	**69**	ENSG00000110318	CEP126	13	2.050272	573.1415	0.005
**20**	ENSG00000079841	RIMS1	20	2.8871886	167.7090838	0.005	**70**	ENSG00000187391	MAGI2	13	1.521818	758.3181	0.007042
**21**	ENSG00000029534	ANK1	20	1.9194571	353.1505697	0.005	**71**	ENSG00000257923	CUX1	13	0.139866	834.1962	0.04712
**22**	ENSG00000197386	HTT	20	0.2950797	1731.169706	0.015384615	**72**	ENSG00000153187	HNRNPU	13	0.087949	2617.038	0.047872
**23**	ENSG00000147133	TAF1	20	0.1471001	475.7077653	0.03	**73**	ENSG00000124181	PLCG1	13	-0.16565	-987.474	0.045
**24**	ENSG00000078018	MAP2	20	-0.6292487	-1053.300005	0.045	**74**	ENSG00000177189	RPS6KA3	13	-0.18935	-493.11	0.031414
**25**	ENSG00000188153	COL4A5	19	0.8177071	1784.805803	0.023668639	**75**	ENSG00000137574	TGS1	13	-0.22269	-457.541	0.025478
**26**	ENSG00000197565	COL4A6	19	0.791811	332.981494	0.005	**76**	ENSG00000071054	MAP4K4	13	-0.29077	-2902.73	0.005882
**27**	ENSG00000150995	ITPR1	19	0.6456234	2273.012262	0.008928571	**77**	ENSG00000185002	RFX6	12	6.27768	38.39665	0.005
**28**	ENSG00000107186	MPDZ	19	0.3118037	515.1317851	0.045	**78**	ENSG00000182255	KCNA4	12	1.9773	3.161625	0.005319
**29**	ENSG00000082898	XPO1	19	-0.1049114	-1306.688358	0.035294118	**79**	ENSG00000152578	GRIA4	12	1.293337	67.66991	0.005
**30**	ENSG00000054523	KIF1B	19	-0.226935	-1109.0336	0.005	**80**	ENSG00000196482	ESRRG	12	1.091739	409.1098	0.005
**31**	ENSG00000107485	GATA3	18	1.0981396	22978.70114	0.015384615	**81**	ENSG00000132326	PER2	12	0.486499	825.0866	0.005
**32**	ENSG00000012048	BRCA1	18	0.2629869	260.9019308	0.04	**82**	ENSG00000152822	GRM1	12	0.474695	3.866918	0.04
**33**	ENSG00000115524	SF3B1	18	-0.1040036	-3384.107714	0.031914894	**83**	ENSG00000135503	ACVR1B	12	0.36676	1082.627	0.005
**34**	ENSG00000081052	COL4A4	18	-0.4677739	-124.3945248	0.04	**84**	ENSG00000172493	AFF1	12	0.344219	1472.818	0.009091
**35**	ENSG00000147255	IGSF1	17	8.8228256	1990.781881	0.005	**85**	ENSG00000129675	ARHGEF6	12	0.332909	816.788	0.005
**36**	ENSG00000163629	PTPN13	17	0.5507444	1118.496508	0.010471204	**86**	ENSG00000112659	CUL9	12	0.322774	636.9331	0.005
**37**	ENSG00000174469	CNTNAP2	17	0.5178737	769.0563081	0.017751479	**87**	ENSG00000064393	HIPK2	12	0.249549	1249.756	0.015
**38**	ENSG00000100393	EP300	17	0.1786484	1169.391119	0.029411765	**88**	ENSG00000108654	DDX5	12	0.242699	8782.769	0.011765
**39**	ENSG00000102974	CTCF	17	0.1075777	443.2205111	0.015706806	**89**	ENSG00000166888	STAT6	12	0.229973	1760.935	0.015385
**40**	ENSG00000153201	RANBP2	17	-0.1714609	-2407.663971	0.005882353	**90**	ENSG00000080503	SMARCA2	12	0.210962	1559.816	0.015385
**41**	ENSG00000132694	ARHGEF11	17	-0.1950456	-1215.689757	0.031914894	**91**	ENSG00000100813	ACIN1	12	0.162443	1385.35	0.015385
**42**	ENSG00000169031	COL4A3	17	-0.557856	-72.3796411	0.02	**92**	ENSG00000099991	CABIN1	12	0.144197	673.7039	0.010471
**43**	ENSG00000178568	ERBB4	16	6.8297983	2850.701131	0.00591716	**93**	ENSG00000169057	MECP2	12	0.12399	498.4853	0.046154
**44**	ENSG00000049759	NEDD4L	16	0.7264956	2316.104497	0.005	**94**	ENSG00000080839	RBL1	12	-0.26069	-359.731	0.041176
**45**	ENSG00000198932	GPRASP1	16	0.5880295	174.6050246	0.005	**95**	ENSG00000147010	SH3KBP1	12	-0.35683	-1271.73	0.005882
**46**	ENSG00000164692	COL1A2	16	0.2062732	50086.72653	0.045	**96**	ENSG00000077092	RARB	12	-0.36704	-215.868	0.045
**47**	ENSG00000159140	SON	16	0.1540387	2496.482491	0.015	**97**	ENSG00000075651	PLD1	12	-0.45916	-699.089	0.005
**48**	ENSG00000167306	MYO5B	16	-0.2420334	-1791.411066	0.03	**98**	ENSG00000105976	MET	12	-0.57089	-1910.4	0.005
**49**	ENSG00000173898	SPTBN2	16	-0.4425482	-2951.055488	0.030769231	**99**	ENSG00000164404	GDF9	11	10.41856	652.5001	0.005
**50**	ENSG00000058335	RASGRF1	16	-0.6225161	-287.1562633	0.047058824	**100**	ENSG00000044524	EPHA3	11	6.127484	449.9175	0.04

Supplementary table 14: TCGA-BRCA DEGs between normal and cancer samples. The full result of the DEG analysis contains 6156 DEGs. For this reason, it is not included here. It can be found in ***Supplementary/ supplementary table 14 - TCGA-BRCA DEGs.csv*** at github.com/AndresMCB/DynamicCancerDriver. Alternatively, it can be accessed at https://doi.org/10.6084/m9.figshare.20448384

Supplementary table 15: DCD.HER2noDEG. List with the DCDs inferred by using "HER2time(Bulk)" that are in the top 100 (ranked by frequency of mutation in TCGA (descending order)) and are not differentially expressed between the conditions normal/cancer. We have included 2 flags indicating whether the DCD gene is a CGC gene/ a CGC Breast Cancer Driver.

	Ensembl.ID	HGNC.symbol	is.CGC	is.BRCA_CGC		Ensembl.ID	HGNC.symbol	is.CGC	is.BRCA_CGC
1	ENSG00000121879	PIK3CA	TRUE	TRUE	34	ENSG00000081052	COL4A4	FALSE	FALSE
2	ENSG00000039068	CDH1	TRUE	TRUE	35	ENSG00000064012	CASP8	TRUE	TRUE
3	ENSG00000141027	NCOR1	TRUE	TRUE	36	ENSG00000131626	PPFIA1	FALSE	FALSE
4	ENSG00000127603	MACF1	FALSE	FALSE	37	ENSG00000147255	IGSF1	FALSE	FALSE
5	ENSG00000081479	LRP2	FALSE	FALSE	38	ENSG00000018236	CNTN1	FALSE	FALSE
6	ENSG00000085224	ATRX	TRUE	FALSE	39	ENSG00000112282	MED23	FALSE	FALSE
7	ENSG00000196712	NF1	TRUE	FALSE	40	ENSG00000170776	AKAP13	FALSE	FALSE
8	ENSG00000166147	FBN1	FALSE	FALSE	41	ENSG00000197081	IGF2R	FALSE	FALSE
9	ENSG00000171862	PTEN	TRUE	FALSE	42	ENSG00000100393	EP300	TRUE	TRUE
10	ENSG00000111642	CHD4	TRUE	FALSE	43	ENSG00000102974	CTCF	TRUE	TRUE
11	ENSG00000065559	MAP2K4	TRUE	TRUE	44	ENSG00000132694	ARHGEF11	FALSE	FALSE
12	ENSG00000142208	AKT1	TRUE	TRUE	45	ENSG00000153201	RANBP2	TRUE	FALSE
13	ENSG00000104517	UBR5	TRUE	FALSE	46	ENSG00000178568	ERBB4	TRUE	FALSE
14	ENSG00000149311	ATM	TRUE	FALSE	47	ENSG00000198753	PLXNB3	FALSE	FALSE
15	ENSG00000178209	PLEC	FALSE	FALSE	48	ENSG00000167306	MYO5B	FALSE	FALSE
16	ENSG00000178104	PDE4DIP	TRUE	FALSE	49	ENSG00000197694	SPTAN1	FALSE	FALSE
17	ENSG00000118058	KMT2A	TRUE	FALSE	50	ENSG00000114127	XRN1	FALSE	FALSE
18	ENSG00000130396	AFDN	TRUE	FALSE	51	ENSG00000049759	NEDD4L	FALSE	FALSE
19	ENSG00000278540	ACACA	FALSE	FALSE	52	ENSG00000196313	POM121	FALSE	FALSE
20	ENSG00000142798	HSPG2	FALSE	FALSE	53	ENSG00000169855	ROBO1	FALSE	FALSE
21	ENSG00000137076	TLN1	FALSE	FALSE	54	ENSG00000183454	GRIN2A	TRUE	FALSE
22	ENSG00000167522	ANKRD11	FALSE	FALSE	55	ENSG00000155511	GRIA1	FALSE	FALSE
23	ENSG00000196367	TRRAP	TRUE	FALSE	56	ENSG00000132849	PATJ	FALSE	FALSE
24	ENSG00000159216	RUNX1	TRUE	FALSE	57	ENSG00000151276	MAGI1	FALSE	FALSE
25	ENSG00000134250	NOTCH2	TRUE	FALSE	58	ENSG00000130985	UBA1	FALSE	FALSE
26	ENSG00000029534	ANK1	TRUE	FALSE	59	ENSG00000196083	IL1RAP	FALSE	FALSE
27	ENSG00000001626	CFTR	FALSE	FALSE	60	ENSG00000197535	MYO5A	TRUE	FALSE
28	ENSG00000138162	TACC2	FALSE	FALSE	61	ENSG00000182752	PAPPA	FALSE	FALSE
29	ENSG00000142949	PTPRF	FALSE	FALSE	62	ENSG00000106571	GLI3	FALSE	FALSE
30	ENSG00000197386	HTT	FALSE	FALSE	63	ENSG00000100852	ARHGAP5	TRUE	FALSE
31	ENSG00000225830	ERCC6	FALSE	FALSE	64	ENSG00000124151	NCOA3	FALSE	FALSE
32	ENSG00000150995	ITPR1	FALSE	FALSE	65	ENSG00000112851	ERBIN	FALSE	FALSE
33	ENSG00000188153	COL4A5	FALSE	FALSE					

Supplementary table 16: DCD.VIMnoDEG. List with the DCDs inferred by using "VIMtime(Bulk)" that are in the top 100 (ranked by frequency of mutation in TCGA (descending order)) and are not differentially expressed between the conditions normal/cancer. We have included 2 flags indicating whether the DCD gene is a CGC gene/ a CGC Breast Cancer Driver.

	Ensembl.ID	HGNC.symbol	is.CGC	is.BRCA_CGC		Ensembl.ID	HGNC.symbol	is.CGC	is.BRCA_CGC
1	ENSG00000121879	PIK3CA	TRUE	TRUE	31	ENSG00000225830	ERCC6	FALSE	FALSE
2	ENSG00000198626	RYR2	FALSE	FALSE	32	ENSG00000070961	ATP2B1	FALSE	FALSE
3	ENSG00000163554	SPTA1	FALSE	FALSE	33	ENSG00000139687	RB1	TRUE	TRUE
4	ENSG00000039068	CDH1	TRUE	TRUE	34	ENSG00000138162	TACC2	FALSE	FALSE
5	ENSG00000127603	MACF1	FALSE	FALSE	35	ENSG00000198646	NCOA6	FALSE	FALSE
6	ENSG00000081479	LRP2	FALSE	FALSE	36	ENSG00000142949	PTPRF	FALSE	FALSE
7	ENSG00000085224	ATRX	TRUE	FALSE	37	ENSG00000188153	COL4A5	FALSE	FALSE
8	ENSG00000127914	AKAP9	TRUE	FALSE	38	ENSG00000137474	MYO7A	FALSE	FALSE
9	ENSG00000196712	NF1	TRUE	FALSE	39	ENSG00000082898	XPO1	TRUE	FALSE
10	ENSG00000065526	SPEN	TRUE	FALSE	40	ENSG00000081052	COL4A4	FALSE	FALSE
11	ENSG00000166147	FBN1	FALSE	FALSE	41	ENSG00000067369	TP53BP1	FALSE	FALSE
12	ENSG00000111642	CHD4	TRUE	FALSE	42	ENSG00000131626	PPFIA1	FALSE	FALSE
13	ENSG00000166963	MAP1A	FALSE	FALSE	43	ENSG00000094631	HDAC6	FALSE	FALSE
14	ENSG00000065559	MAP2K4	TRUE	TRUE	44	ENSG00000147162	OGT	FALSE	FALSE
15	ENSG00000142208	AKT1	TRUE	TRUE	45	ENSG00000147255	IGSF1	FALSE	FALSE
16	ENSG00000149311	ATM	TRUE	FALSE	46	ENSG00000163629	PTPN13	TRUE	FALSE
17	ENSG00000178209	PLEC	FALSE	FALSE	47	ENSG00000018236	CNTN1	FALSE	FALSE
18	ENSG00000134982	APC	TRUE	FALSE	48	ENSG00000169031	COL4A3	FALSE	FALSE
19	ENSG00000118058	KMT2A	TRUE	FALSE	49	ENSG00000100393	EP300	TRUE	TRUE
20	ENSG00000142798	HSPG2	FALSE	FALSE	50	ENSG00000102974	CTCF	TRUE	TRUE
21	ENSG00000100503	NIN	TRUE	FALSE	51	ENSG00000112282	MED23	FALSE	FALSE
22	ENSG00000130396	AFDN	TRUE	FALSE	52	ENSG00000153201	RANBP2	TRUE	FALSE
23	ENSG00000096433	ITPR3	FALSE	FALSE	53	ENSG00000101868	POLA1	FALSE	FALSE
24	ENSG00000196367	TRRAP	TRUE	FALSE	54	ENSG00000197081	IGF2R	FALSE	FALSE
25	ENSG00000167522	ANKRD11	FALSE	FALSE	55	ENSG00000170776	AKAP13	FALSE	FALSE
26	ENSG00000137076	TLN1	FALSE	FALSE	56	ENSG00000198753	PLXNB3	FALSE	FALSE
27	ENSG00000159216	RUNX1	TRUE	FALSE	57	ENSG00000197694	SPTAN1	FALSE	FALSE
28	ENSG00000134250	NOTCH2	TRUE	FALSE	58	ENSG00000114127	XRN1	FALSE	FALSE
29	ENSG00000029534	ANK1	TRUE	FALSE	59	ENSG00000167306	MYO5B	FALSE	FALSE
30	ENSG00000001626	CFTR	FALSE	FALSE	60	ENSG00000085511	MAP3K4	FALSE	FALSE

Supplementary table 17: dynamic cancer drivers inferred from single cell data when using Monocle3 pseudotime (event detection using HER2), ranked by Relative Causal Impact (descending order). A full comprehensive list (687 DCDs) can be found in ***Supplementary/ supplementary table 17 - DCDs(HER2)monocle3_SC.csv*** at github.com/AndresMCB/DynamicCancerDriver. Alternatively, it can be accessed at https://doi.org/10.6084/m9.figshare.20448384

	Ensembl.ID	HGNC.symbol	RelEffect	AbsEffect	p		Ensembl.ID	HGNC.symbol	RelEffect	AbsEffect	p
**1**	ENSG00000026025	VIM	5497.015677	48.46322256	0.005	**51**	ENSG00000112242	E2F3	59.01830337	5.408361	0.005464481
**2**	ENSG00000150907	FOXO1	4714.552873	5.608519119	0.005	**52**	ENSG00000076003	MCM6	54.98556671	11.11409	0.005319149
**3**	ENSG00000131981	LGALS3	4144.929251	84.26206587	0.005319149	**53**	ENSG00000169679	BUB1	54.42728252	5.304895	0.005
**4**	ENSG00000090104	RGS1	2903.345228	87.32849747	0.005464481	**54**	ENSG00000011243	AKAP8L	47.46843703	36.62557	0.005235602
**5**	ENSG00000146648	EGFR	2355.218769	6.286198263	0.005	**55**	ENSG00000149269	PAK1	46.11539601	30.73035	0.005319149
**6**	ENSG00000007372	PAX6	2125.433108	9.776856375	0.005464481	**56**	ENSG00000077235	GTF3C1	43.64561452	12.07654	0.005464481
**7**	ENSG00000139618	BRCA2	1929.235161	10.1325149	0.005464481	**57**	ENSG00000150995	ITPR1	42.72549849	11.15117	0.005
**8**	ENSG00000163629	PTPN13	1432.959532	9.865542452	0.005319149	**58**	ENSG00000076984	MAP2K7	40.18901772	0.720771	0.005
**9**	ENSG00000113580	NR3C1	1331.273657	18.72189155	0.005464481	**59**	ENSG00000158716	DUSP23	37.49162824	20.19945	0.005464481
**10**	ENSG00000170312	CDK1	1324.514951	38.18243465	0.005	**60**	ENSG00000118785	SPP1	36.6437301	60.92617	0.005464481
**11**	ENSG00000145675	PIK3R1	1268.243844	43.10251887	0.005	**61**	ENSG00000130066	SAT1	34.5814983	8263.452	0.005464481
**12**	ENSG00000204388	HSPA1B	875.1034225	82.62241402	0.005464481	**62**	ENSG00000185591	SP1	33.33557001	13.55126	0.005
**13**	ENSG00000147168	IL2RG	873.7077514	24.32384305	0.005464481	**63**	ENSG00000115414	FN1	31.9640872	53.38424	0.005
**14**	ENSG00000115170	ACVR1	866.3055373	11.72843566	0.005464481	**64**	ENSG00000126351	THRA	30.44203132	7.15252	0.005464481
**15**	ENSG00000065534	MYLK	863.2676005	19.83828168	0.005464481	**65**	ENSG00000175899	A2M	30.00699549	128.5976	0.005
**16**	ENSG00000116478	HDAC1	849.4868708	20.04177936	0.005	**66**	ENSG00000101224	CDC25B	29.95894139	8.40489	0.005
**17**	ENSG00000008710	PKD1	678.7199206	1.631221216	0.005	**67**	ENSG00000131323	TRAF3	29.22687329	2.972127	0.005
**18**	ENSG00000057657	PRDM1	645.531052	8.050021523	0.005	**68**	ENSG00000101868	POLA1	26.83742502	4.764538	0.005464481
**19**	ENSG00000134954	ETS1	385.4317008	3.463086243	0.005	**69**	ENSG00000089685	BIRC5	26.67220914	20.99904	0.021857923
**20**	ENSG00000161638	ITGA5	364.1595487	9.65155461	0.005235602	**70**	ENSG00000106799	TGFBR1	25.68393348	19.54363	0.005
**21**	ENSG00000105810	CDK6	327.1609314	10.69510869	0.005	**71**	ENSG00000125817	CENPB	25.27345499	0.877761	0.005319149
**22**	ENSG00000211899	IGHM	296.2099599	5.988681984	0.005	**72**	ENSG00000173020	GRK2	25.05807981	1.365911	0.005
**23**	ENSG00000162511	LAPTM5	295.1172371	14.75063261	0.005464481	**73**	ENSG00000111445	RFC5	24.7227333	14.36336	0.005
**24**	ENSG00000120899	PTK2B	283.5072807	5.138283775	0.005	**74**	ENSG00000173757	STAT5B	23.72027886	6.133278	0.005
**25**	ENSG00000130699	TAF4	270.6514607	3.524711563	0.005	**75**	ENSG00000117394	SLC2A1	23.65214494	37.57094	0.005025126
**26**	ENSG00000061273	HDAC7	256.0360489	7.112382978	0.005464481	**76**	ENSG00000116809	ZBTB17	23.09264935	16.5387	0.005319149
**27**	ENSG00000171425	ZNF581	250.9541922	10.09179279	0.005	**77**	ENSG00000196482	ESRRG	21.97905737	12.7691	0.005464481
**28**	ENSG00000165392	WRN	228.0468516	9.058240204	0.005235602	**78**	ENSG00000185499	MUC1	21.6742046	260.7841	0.005
**29**	ENSG00000071539	TRIP13	223.1553307	4.448673093	0.005464481	**79**	ENSG00000129245	FXR2	21.58980148	6.144121	0.005319149
**30**	ENSG00000169554	ZEB2	210.4185352	7.532648196	0.005	**80**	ENSG00000171223	JUNB	21.16142238	19.90677	0.005
**31**	ENSG00000146232	NFKBIE	201.0148845	8.677575516	0.005464481	**81**	ENSG00000169375	SIN3A	20.91266456	65.02031	0.005
**32**	ENSG00000182195	LDOC1	194.4252793	34.65212748	0.005	**82**	ENSG00000072201	LNX1	20.77137243	13.89379	0.005235602
**33**	ENSG00000086015	MAST2	186.2171539	4.115568991	0.005319149	**83**	ENSG00000132002	DNAJB1	20.52972325	130.2722	0.005464481
**34**	ENSG00000157933	SKI	185.711374	1.03574966	0.005319149	**84**	ENSG00000072062	PRKACA	20.45013863	3.467959	0.005
**35**	ENSG00000080839	RBL1	160.4416156	5.529130287	0.005464481	**85**	ENSG00000143622	RIT1	19.37067322	61.68632	0.005464481
**36**	ENSG00000163565	IFI16	136.502815	24.41421937	0.005	**86**	ENSG00000108262	GIT1	18.86727341	4.247711	0.005319149
**37**	ENSG00000100297	MCM5	123.7222159	8.307642107	0.005464481	**87**	ENSG00000168395	ING5	18.19410268	6.602726	0.005
**38**	ENSG00000113140	SPARC	121.0313529	13.42570679	0.005	**88**	ENSG00000149257	SERPINH1	17.99791167	6.785631	0.005
**39**	ENSG00000103168	TAF1C	109.1031238	2.267694096	0.005	**89**	ENSG00000153487	ING1	17.61159174	5.329368	0.005
**40**	ENSG00000100365	NCF4	103.6331154	2.048389558	0.005464481	**90**	ENSG00000140564	FURIN	17.05784999	3.395597	0.005
**41**	ENSG00000110031	LPXN	101.9555336	10.79265484	0.005	**91**	ENSG00000141646	SMAD4	16.78768469	13.59412	0.005
**42**	ENSG00000123609	NMI	100.1228912	17.83350199	0.005	**92**	ENSG00000067369	TP53BP1	16.07849707	33.25126	0.005235602
**43**	ENSG00000079819	EPB41L2	97.43900444	13.96368337	0.005464481	**93**	ENSG00000136527	TRA2B	15.85084817	221.5867	0.005
**44**	ENSG00000094804	CDC6	95.56694704	54.24212624	0.005464481	**94**	ENSG00000150760	DOCK1	15.81835072	7.598781	0.005
**45**	ENSG00000181191	PJA1	92.19887376	10.00677279	0.005319149	**95**	ENSG00000092820	EZR	15.30487631	643.9626	0.021857923
**46**	ENSG00000115904	SOS1	87.69865463	19.24214135	0.005235602	**96**	ENSG00000117632	STMN1	15.25582873	324.2975	0.005464481
**47**	ENSG00000204389	HSPA1A	76.13635005	251.2691652	0.005464481	**97**	ENSG00000111348	ARHGDIB	14.79380311	205.5064	0.005464481
**48**	ENSG00000107438	PDLIM1	72.05086323	51.4028857	0.005	**98**	ENSG00000101400	SNTA1	14.60203488	3.230965	0.005464481
**49**	ENSG00000163602	RYBP	67.03034468	8.669466149	0.005	**99**	ENSG00000134899	ERCC5	14.49894363	16.71281	0.005464481
**50**	ENSG00000153094	BCL2L11	59.43233576	18.57503198	0.005	**100**	ENSG00000105229	PIAS4	14.33159882	2.572477	0.005464481

Supplementary table 18: dynamic cancer drivers inferred from single cell data when using Monocle3 pseudotime (event detection using VIM), ranked by Relative Causal Impact (descending order). A full comprehensive list (665 DCDs) can be found in ***Supplementary/ supplementary table 18 - DCDs(VIM)monocle3_SC.csv*** at github.com/AndresMCB/DynamicCancerDriver. Alternatively, it can be accessed at https://doi.org/10.6084/m9.figshare.20448384

	Ensembl.ID	HGNC.symbol	RelEffect	AbsEffect	p		Ensembl.ID	HGNC.symbol	RelEffect	AbsEffect	p
**1**	ENSG00000095066	HOOK2	5167.37962	27.77482041	0.021857923	**51**	ENSG00000171425	ZNF581	37.92648	9.866243113	0.005
**2**	ENSG00000090104	RGS1	3643.090673	87.90355845	0.005464481	**52**	ENSG00000126351	THRA	34.58125	7.225962477	0.005464481
**3**	ENSG00000146648	EGFR	3112.550314	6.327804137	0.005	**53**	ENSG00000101868	POLA1	34.4132	4.833803455	0.005464481
**4**	ENSG00000131981	LGALS3	3005.447105	84.80324928	0.005464481	**54**	ENSG00000106799	TGFBR1	34.32812	19.85816402	0.005
**5**	ENSG00000139618	BRCA2	2429.572877	10.19961297	0.005464481	**55**	ENSG00000101224	CDC25B	33.83564	8.491069067	0.005
**6**	ENSG00000113580	NR3C1	1724.40904	18.84708344	0.005464481	**56**	ENSG00000158092	NCK1	32.89718	37.94398278	0.005
**7**	ENSG00000145675	PIK3R1	1680.035878	43.39169659	0.005	**57**	ENSG00000149269	PAK1	32.82206	30.64050526	0.005235602
**8**	ENSG00000116478	HDAC1	1124.17988	20.17814199	0.005	**58**	ENSG00000072201	LNX1	31.92938	14.21180431	0.005464481
**9**	ENSG00000147168	IL2RG	1098.621453	24.48803595	0.005464481	**59**	ENSG00000008710	PKD1	30.76622	1.590865109	0.005
**10**	ENSG00000115170	ACVR1	1049.61868	11.80722	0.005464481	**60**	ENSG00000116809	ZBTB17	29.1999	16.79221854	0.005464481
**11**	ENSG00000118971	CCND2	987.3905813	5.573443329	0.005464481	**61**	ENSG00000175324	LSM1	28.95422	78.85814772	0.005
**12**	ENSG00000057657	PRDM1	834.2949252	8.105301136	0.005	**62**	ENSG00000196482	ESRRG	27.85875	12.97142189	0.005464481
**13**	ENSG00000129675	ARHGEF6	821.780364	6.617159502	0.005464481	**63**	ENSG00000011243	AKAP8L	27.61783	36.30622058	0.005
**14**	ENSG00000204388	HSPA1B	770.6368303	83.14673187	0.005464481	**64**	ENSG00000153487	ING1	26.39598	5.45901815	0.005
**15**	ENSG00000065534	MYLK	752.0981175	19.96368402	0.005464481	**65**	ENSG00000089685	BIRC5	26.19338	21.1218908	0.021857923
**16**	ENSG00000211899	IGHM	499.8789776	6.035970738	0.005	**66**	ENSG00000115904	SOS1	26.06123	18.86429799	0.005235602
**17**	ENSG00000157110	RBPMS	419.6438232	19.04546208	0.005	**67**	ENSG00000158716	DUSP23	25.46537	20.06249137	0.005464481
**18**	ENSG00000105810	CDK6	371.5603146	10.76870491	0.005	**68**	ENSG00000185499	MUC1	24.50736	263.7965821	0.005
**19**	ENSG00000134954	ETS1	340.6730401	3.483910257	0.005	**69**	ENSG00000143622	RIT1	24.07303	62.68753871	0.005464481
**20**	ENSG00000130699	TAF4	329.8696983	3.550019874	0.005	**70**	ENSG00000131323	TRAF3	23.8326	2.968599191	0.005
**21**	ENSG00000162511	LAPTM5	318.33128	14.85038511	0.005464481	**71**	ENSG00000175115	PACS1	23.41073	5.53003604	0.027322404
**22**	ENSG00000169554	ZEB2	305.5653887	7.59290374	0.005	**72**	ENSG00000129245	FXR2	22.4708	6.192903743	0.005319149
**23**	ENSG00000071539	TRIP13	293.700486	4.482457866	0.005464481	**73**	ENSG00000118785	SPP1	20.9329	60.07447256	0.005464481
**24**	ENSG00000123609	NMI	203.4667534	18.03225441	0.005	**74**	ENSG00000137337	MDC1	20.24821	7.172730382	0.005
**25**	ENSG00000086015	MAST2	199.5809937	4.143862595	0.005464481	**75**	ENSG00000132002	DNAJB1	20.11164	130.9474113	0.005464481
**26**	ENSG00000163565	IFI16	194.1566688	24.62645186	0.005	**76**	ENSG00000165392	WRN	19.85458	8.712394816	0.005
**27**	ENSG00000080839	RBL1	190.9648538	5.570665975	0.005464481	**77**	ENSG00000111276	CDKN1B	18.5527	7.665552551	0.005464481
**28**	ENSG00000146232	NFKBIE	173.4138221	8.726712779	0.005464481	**78**	ENSG00000111348	ARHGDIB	18.11962	209.2773074	0.005464481
**29**	ENSG00000113140	SPARC	165.9340664	13.54320287	0.005	**79**	ENSG00000149257	SERPINH1	17.90422	6.822094239	0.005
**30**	ENSG00000079819	EPB41L2	165.0299126	14.11337242	0.005464481	**80**	ENSG00000149554	CHEK1	17.80112	22.87938021	0.021857923
**31**	ENSG00000126561	STAT5A	158.1172205	7.212139828	0.005	**81**	ENSG00000140939	NOL3	17.65267	11.17505869	0.005
**32**	ENSG00000061273	HDAC7	156.6370247	7.138336323	0.005464481	**82**	ENSG00000150907	FOXO1	17.48704	5.332548906	0.005
**33**	ENSG00000100297	MCM5	155.7528143	8.375573751	0.005464481	**83**	ENSG00000125817	CENPB	17.30969	0.867197468	0.005319149
**34**	ENSG00000100365	NCF4	135.1333586	2.066337565	0.005464481	**84**	ENSG00000114107	CEP70	17.13509	34.15585737	0.02
**35**	ENSG00000094804	CDC6	131.2079871	54.74898538	0.005464481	**85**	ENSG00000177606	JUN	16.87409	24.06345225	0.005
**36**	ENSG00000175899	A2M	129.6239742	132.7250018	0.005	**86**	ENSG00000140396	NCOA2	16.59145	10.73908573	0.005464481
**37**	ENSG00000110031	LPXN	110.1277824	10.87080061	0.005	**87**	ENSG00000067369	TP53BP1	15.95731	33.372974	0.005464481
**38**	ENSG00000077235	GTF3C1	99.70110306	12.30827464	0.005464481	**88**	ENSG00000132964	CDK8	15.8527	11.8504762	0.005464481
**39**	ENSG00000157933	SKI	98.25606218	1.037325369	0.005319149	**89**	ENSG00000007372	PAX6	15.03364	9.211477627	0.021857923
**40**	ENSG00000204389	HSPA1A	97.73041504	253.6317525	0.005464481	**90**	ENSG00000163629	PTPN13	13.68312	9.239446515	0.005
**41**	ENSG00000181191	PJA1	96.41729107	10.07669394	0.005464481	**91**	ENSG00000198793	MTOR	13.51035	10.26927655	0.021857923
**42**	ENSG00000107438	PDLIM1	94.04056497	51.90389966	0.005	**92**	ENSG00000104765	BNIP3L	13.41124	108.5863932	0.005235602
**43**	ENSG00000076003	MCM6	73.13006448	11.23628687	0.005464481	**93**	ENSG00000155657	TTN	13.2287	1.290281094	0.005464481
**44**	ENSG00000169679	BUB1	72.33922917	5.36341446	0.005	**94**	ENSG00000135723	FHOD1	13.0542	12.15724464	0.005
**45**	ENSG00000120899	PTK2B	71.11948886	5.118036096	0.005	**95**	ENSG00000147044	CASK	12.74202	45.51641543	0.005464481
**46**	ENSG00000182195	LDOC1	64.67195828	34.5234399	0.005	**96**	ENSG00000124357	NAGK	12.05479	69.43220162	0.005
**47**	ENSG00000141736	ERBB2	53.78312395	100.8353444	0.005	**97**	ENSG00000204469	PRRC2A	12.02893	5.142489858	0.005464481
**48**	ENSG00000150995	ITPR1	53.25922334	11.27481766	0.005	**98**	ENSG00000115414	FN1	11.83336	50.99172744	0.005
**49**	ENSG00000076984	MAP2K7	43.35798709	0.726756156	0.005	**99**	ENSG00000169375	SIN3A	11.76968	63.0743067	0.005
**50**	ENSG00000136527	TRA2B	40.73948841	230.7600479	0.005	**100**	ENSG00000091073	DTX2	11.60333	6.540285256	0.021857923
